# Centromeric SMC1 promotes centromere clustering and stabilizes meiotic homolog pairing

**DOI:** 10.1371/journal.pgen.1008412

**Published:** 2019-10-14

**Authors:** Talia Hatkevich, Vincent Boudreau, Thomas Rubin, Paul S. Maddox, Jean-René Huynh, Jeff Sekelsky

**Affiliations:** 1 Curriculum in Genetics and Molecular Biology, University of North Carolina, Chapel Hill, North Carolina, United States of America; 2 Department of Biology, University of North Carolina, Chapel Hill, North Carolina, United States of America; 3 CIRB, Collège de France, PSL Research University, CNRS UMR7241, Inserm U1050, Paris, France; 4 Integrative Program in Biological and Genome Sciences, University of North Carolina, Chapel Hill, North Carolina, United States of America; Stanford University School of Medicine, UNITED STATES

## Abstract

During meiosis, each chromosome must selectively pair and synapse with its own unique homolog to enable crossover formation and subsequent segregation. How homolog pairing is maintained in early meiosis to ensure synapsis occurs exclusively between homologs is unknown. We aimed to further understand this process by examining the meiotic defects of a unique *Drosophila* mutant, *Mcm5*^*A7*^. We found that *Mcm5*^*A7*^ mutants are proficient in homolog pairing at meiotic onset yet fail to maintain pairing as meiotic synapsis ensues, causing seemingly normal synapsis between non-homologous loci. This pairing defect corresponds with a reduction of SMC1-dependent centromere clustering at meiotic onset. Overexpressing SMC1 in this mutant significantly restores centromere clustering, homolog pairing, and crossover formation. These data indicate that the initial meiotic pairing of homologs is not sufficient to yield synapsis exclusively between homologs and provide a model in which meiotic homolog pairing must be stabilized by centromeric SMC1 to ensure proper synapsis.

## Author summary

Sexually reproducing organisms must produce gametes (sperm and eggs) that have one copy of each chromosome. This is accomplished through a special cell division called meiosis. Each chromosome replicates to generate identical sister chromatids, then finds and pairs with its unique partner chromosome. A well-regulated recombination process then generates crossovers between paired maternal/paternal partners; these crossovers ensure accurate chromosome segregation in meiosis. The pairing process is very poorly understood. The *Drosophila melanogaster* (fruit fly) *Mcm5*^*A7*^ mutation was previously shown to reduce crossovers but we show here that this is due to defects in meiotic chromosome pairing. We trace the primary defect to failure to load cohesins, which hold sister chromatids together but have additional roles in meiosis, at the centromere–the region that will later direct chromosome segregation. Thus, defects in centromeric cohesion lead to loss of chromosome pairing and loss of recombination along the arms of the chromosomes, and ultimately loss of fidelity during chromosome segregation.

## Introduction

**Fig 1:**
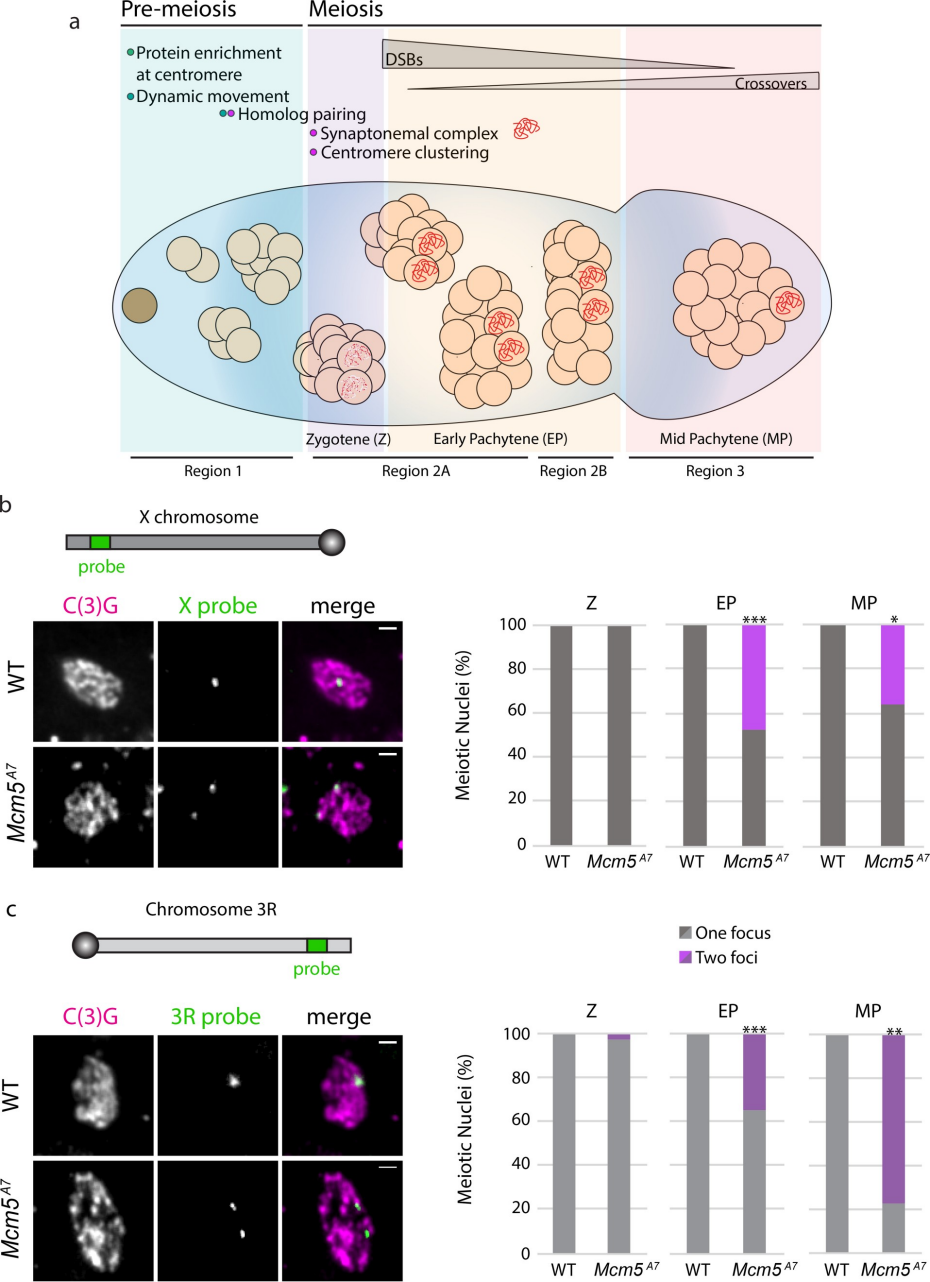
Meiotic pairing is perturbed in *Mcm5*^*A7*^ mutants. a. Schematic depiction of the *Drosophila* germarium. At the anterior portion (the pre-meiotic region, Region 1), the germline stem cell (brown cell) divides to yield a cytoblast, which undergoes four subsequent rounds of division to yield a 16-cell cyst. In the pre-meiotic region, proteins, such as SMC1 and C(3)G, are enriched at the centromeres, and within the 8-cell cyst, chromosomes exhibit centromere-direct rapid movements. Within the first 16-cell cyst (zygotene; Region 2A), homologous chromosomes pair, centromeres cluster into 1 or 2 groups, and up to four cells initiate meiosis, expressing patches of SC (red dots). As the 16-cell cyst enters early pachytene (EP) (Region 2A), only two continue as pro-oocytes to form full length synaptonemal complex (red). Meiotic double-strand breaks (DSBs) are formed and repaired via homologous recombination (HR) throughout the germarium’s posterior (Regions 2A, 2B) to yield noncrossover and crossover products. At the most posterior tip, signifying mid-pachytene (MP), only one cell within the cyst has been selected to become the oocyte, and all DSBs are repaired. Cytological regions of the germarium (Region 1, 2A, 2B, and 3) are depicted. b. Top: schematic of *X* chromosome and relative location of *X*-probe, not drawn to scale. Left: Representative images paired (*WT*) and unpaired (*Mcm5*^*A7*^) *X*-probes (green) in meiotic cells, indicated by C(3)G expression (magenta). Images are of meiotic nuclei in Region 2A. Scale bar = 1 μm. Right: Quantification of percent paired and unpaired nuclei in *WT* and *Mcm5*^*A7*^ in Z (*WT n =* 33, *Mcm5*^*A7*^ = 32), EP (*WT n =* 130, *Mcm5*^*A7*^ = 118; ***p < 0.0001, chi-square), and MP (*WT n =* 10, *Mcm5*^*A7*^ = 11; *p = 0.01, chi-square). c. Top: schematic of the right arm of chromosome *3* (*3*R) and relative location of *3*R-probe, not drawn to scale. Left: Representative images paired (*WT*) and unpaired (*Mcm5*^*A7*^) *3*R-probes (green) in meiotic cells, represented by C(3)G expression (magenta). *WT* image is of 2A nucleus, *Mcm5*^*A7*^ is of Region 3 nucleus. Right: Quantification of percent paired and unpaired nuclei in *WT* and *Mcm5*^*A7*^ in Z (*WT n =* 37, *Mcm5*^*A7*^ = 33), EP (*WT n =* 104, *Mcm5*^*A7*^ = 97; ***p < 0.0001, chi-square), and MP (*WT n =* 10, *Mcm5*^*A7*^ = 9; **p = 0.0066, chi-square). Brightness, contrast, and texture (smoothed) of images have been adjusted for clarity.

Accurate segregation of homologous chromosomes during the first meiotic division is essential to reestablish the diploid genome upon sexual fertilization. To ensure faithful meiosis I chromosomal segregation, homologs must become physically connected in part through crossover formation. To enable homolog crossover events, a series of chromosomal and cellular events occur in early meiotic prophase I [1] (Fig 1A).

During or just prior to the onset of meiosis, homologous chromosomes pair along their entire lengths [2]. Between paired homologs, synapsis, the formation of the synaptonemal complex (SC), ensues. The SC is a tripartite scaffold built between homologs extending the length of the chromosomes and consists of a central region that is nestled between two lateral elements, which are successors of cohesin-based chromosome axes formed between sister chromatids. Coincident with synapsis, DSBs are formed and repaired using a homologous template via homologous recombination (HR), resulting in crossover formation between homologs [3].

Perhaps the most enigmatic event within early meiosis is the mechanism by which a meiotic chromosome selectively pairs and synapses with its unique homologous partner. Initial homolog pairing is believed to be facilitated through early meiotic chromosome movement and telomere or the centromere clustering [reviewed in 2, 4, 5]. However, how homologous pairing is maintained during synapsis to ensure the SC is formed exclusively between homologs is unknown.

The model organism *Drosophila melanogaster* has been used to uncover meiotic mechanisms for over a century [6]. In *Drosophila*, prior to meiosis, chromosomes enter the germline unpaired (Fig 1A); throughout the pre-meiotic region, homologous chromosomes gradually pair. In the nuclei at the last mitotic division prior to meiotic onset (in the 8-cell cyst), centromere-directed chromosomal movements occur, presumably ensuring complete homologous pairing [7, 8]. Also during pre-meiotic mitotic cycles, several proteins, including the cohesin SMC1, are enriched at the centromere [9, 10]. The onset of meiotic prophase I occurs in the 16-cell cyst. At zygotene, the first cytologically resolved stage of prophase, centromeres are clustered into 1 or 2 groups [11], and the SC nucleates in patches along chromosome arms [12]. As zygotene proceeds into early pachytene, the SC extends between paired chromosomes, yielding full-length SC exclusively between homologs. How these early meiotic events, particularly SMC1 enrichment at the centromere and centromere clustering, contribute to meiotic homologous pairing and synapsis in *Drosophila* is largely unknown.

In this study, we used the *Drosophila* early meiotic program and a unique genetic mutant to investigate how homolog pairing is maintained during meiotic synapsis. We discovered that meiotic homologs in a previously described *Drosophila* mutant, *Mcm5*^*A7*^ [1], initially pair, but are unable to maintain pairing during synapsis, suggesting that initial meiotic pairing must be subsequently stabilized by an unknown mechanism to ensure proper synapsis. Using *Mcm5*^*A7*^ as a genetic tool to interrogate pairing stabilization mechanism(s), we find that SMC1 localization at the centromere is compromised, correlating with a severe defect in meiotic centromere clustering and a decrease of crossover formation. However, arm cohesion and SC structure appear unperturbed in these mutants. By overexpressing SMC1, we show that the defects in centromere clustering, meiotic homolog pairing, homosynapsis, and crossing over in *Mcm5*^*A7*^ mutants are caused by a lack of centromeric SMC1 localization at meiotic onset. From our results, we suggest a model for proper synapsis in which initial meiotic pairing must be stabilized by centromere clustering, a meiotic event produced by SMC1-enrichment at the centromere and dynamic chromosome movements.

## Results

### Mcm5^A7^ mutants are proficient in initial meiotic pairing but deficient in maintenance of pairing

The *Mcm5*^*A7*^ allele, discovered in a screen for *Drosophila* meiotic mutants, is a missense mutation that changes an aspartic acid residue to valine; this reside is C-terminal to the AAA^+^ ATPase domain but is conserved among Mcm5 orthologs but not other MCM paralogs [1]. *Mcm5*^*A7*^ mutants have an *X-*NDJ rate of ~25% that is accompanied with a 90% decrease of crossovers on the *X* chromosome. Interestingly, the SC, as shown through staining of the central region protein C(3)G, appears normal, and DSBs are created and repaired with normal kinetics [1]. Why crossovers are severely decreased in *Mcm5*^*A7*^ mutants was unknown at the time of this study.

We hypothesized that a lack of meiotic homolog pairing could result in the severe loss of crossovers in *Mcm5*^*A7*^ mutants. To test this, we examined the frequencies of *X* and chromosome *3R* homolog pairing in zygotene, early pachytene, and mid-pachytene meiotic cells using IF/FISH. Zygotene is the earliest cytologically resolved meiotic stage in the *Drosophila* germarium and is defined by the presence of SC patches in the 16-cell cyst. Early pachytene is defined by full-length SC in the early 16-cell cysts (Region 2A of the germarium), and mid-pachytene is defined as the most posterior nucleus in the germarium that expresses full-length SC (Region 3) [13].

At the examined *X* locus, which spans cytological regions 6E-7B, wild-type meiotic cells exhibit one focus throughout zygotene, early pachytene, and mid-pachytene (Fig 1B). In *Mcm5*^*A7*^ mutants, we observed one focus at 100% frequency in zygotene. Strikingly, we can resolve two foci in approximately half of the nuclei in *Mcm5*^*A7*^ mutants during early pachytene (***p < 0.0001) and mid-pachytene (*p = 0.01).

Similarly, at the *3*R locus wild-type homologous chromosomes are paired at 100% frequency in zygotene, early pachytene, and mid-pachytene (Fig 1C). However, in *Mcm5*^*A7*^ mutants, the homologs of chromosome *3*R in zygotene are paired at nearly 100% frequency, yet we can resolve two *3*R foci in 35% of early pachytene nuclei (***p < 0.0001) and 78% of mid-pachytene nuclei (**p = 0.0002).

These results show that in *Mcm5*^*A7*^ mutants, meiotic chromosomes enter meiosis paired, but as the meiotic nuclei proceed through meiosis, homologous pairing is not maintained. This suggests that initial homolog pairing must be stabilized in early prophase and that this mechanism is perturbed in *Mcm5*^*A7*^ mutants. Therefore, we reasoned that *Mcm5*^*A7*^ can be used as a genetic tool to interrogate the mechanism that stabilizes meiotic pairing.

### The synaptonemal complex shows no observable defects in *Mcm5*^*A7*^ mutants

**Fig 2:**
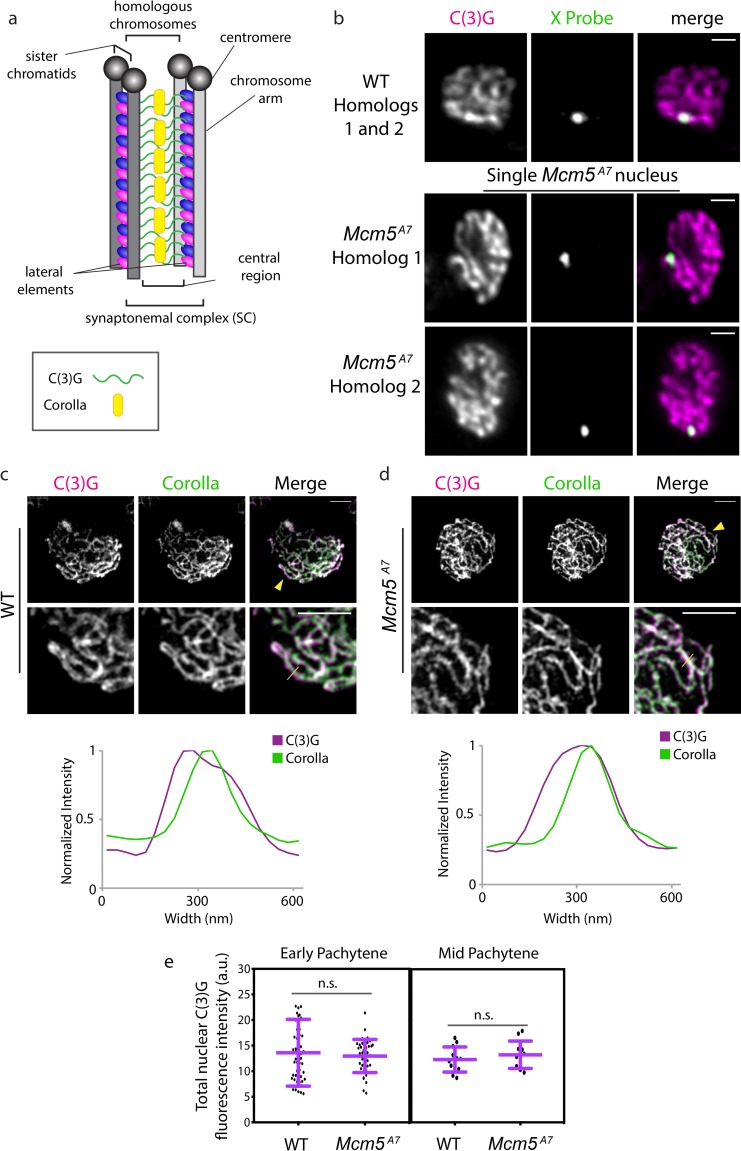
Synaptonemal complex exhibits no observable defects in *Mcm5*^*A7*^ mutants. a. Schematic depiction of SC between two homologous chromosomes. The SC is composed of two lateral elements flanking a central region. The lateral elements are predecessors of the axial element, which is formed between sister chromatids and are composed of two cohesion complexes (blue and pink ovals). The central region consists, in part, of a C(3)G (green) dimer spanning the lateral elements, with pillar proteins such as Corolla (yellow) embedded within the central region. Enrichment of proteins at the centromere is not depicted. b. Super-resolution images of C(3)G and *X*-probe in *WT* (paired) and *Mcm5*^*A7*^ (unpaired) in whole-mount germaria, quantified in Fig 1. The images of *Mcm5*^*A7*^ are of the same nucleus but of different *Z* slices to capture both *X*-probes. Brightness and contrast have been adjusted for clarity. Scale bar = 1 μm. Refer to S1 and S2 Movies. c. Top: Representative image of C(3)G (magenta) and Corolla (green) in a *WT* meiotic chromosome spread. Brightness and contrast have been adjusted for clarity. Yellow arrowhead indicates area magnified in lower panel (middle). Scale bar = 2 μm. Middle: Magnification to detail the localization of C(3)G and Corolla. Scale bar = 2 μm. Yellow line indicates the area quantified for normalized intensity. Bottom: Normalized intensity of C(3)G and Corolla in a representative SC tract to demonstrate localization. d. Top: Representative image of C(3)G (magenta) and Corolla (green) in *Mcm5*^*A7*^ meiotic chromosome spread. Yellow arrowhead indicates area magnified in lower panel (middle). Scale bar = 2 μm. Middle: Magnification to detail the localization of C(3)G and Corolla. Scale bar = 2 μm. Yellow line indicates the area that was quantified for normalized intensity. Bottom: Normalized intensity of C(3)G and Corolla to demonstrate localization. e. Left panel: Quantification of nuclear C(3)G signal at early pachytene in *WT* (*n* = 52) and *Mcm5*^*A7*^ (*n* = 41) meiotic nuclei. *p* = 0.5601, unpaired T-test. Data are represented as mean ± SD. Right panel: Quantification of nuclear C(3)G signal in mid-pachytene in *WT* (*n* = 12) and *Mcm5*^*A7*^ (*n* = 11) meiotic nuclei. *p* = 0.3993, unpaired T-test. Data are represented as mean ± SD. Refer to S1 Fig for images and further analysis.

Although pachytene homolog pairing is disrupted at a high frequency in *Mcm5*^*A7*^ mutants, the SC, as determined by C(3)G staining, still forms [1] (Fig 2A). To explain this, we hypothesize that either (1) the unpaired loci do not correspond with linear SC, or (2) the unpaired loci are forming stable SC with non-homologous loci, creating heterosynapsis. To differentiate between these two, we examined whole mount germaria with IF/FISH and super-resolution microscopy (AIRY Scan) and examined tracts of SC. In wild-type cells, we can discern that one linear tract of C(3)G is built between the paired *X* locus (Fig 2B). In *Mcm5*^*A7*^ mutants, separate homologous loci are associated with separate linear tracts of C(3)G, suggesting that the unpaired *X* loci are synapsed with non-homologous loci (see S1 Movie and S2 Movie). From these data, we conclude that *Mcm5*^*A7*^ mutants have the ability to assemble SC between non-homologous sequences.

To determine the nature of heterosynapsis, we examined the localization of two SC central region proteins, C(3)G and Corolla (Fig 2A). In wild-type, Corolla co-localizes with C(3)G dimers [14], as shown in Fig 2C under structured-illumination microscopy. Under higher resolution, Corolla and C(3)G signal were found to overlap, with C(3)G signal being wider, as expected due to its dimer-dimer conformation [15]. In *Mcm5*^*A7*^ mutants, Corolla and C(3)G exhibit a similar localization pattern (Fig 2D). To assess levels of these central region proteins, we quantified total C(3)G nuclear signal during early and mid-pachytene in wild-type and *Mcm5*^*A7*^ mutants (Fig 2E, S1 Fig). During these timepoints, we see no significant differences between wild-type and *Mcm5*^*A7*^ C(3)G nuclear fluorescence intensity (*p* = 0.5601 and *p* = 0.3993, respectively, unpaired T-test). From our results, we conclude that *Mcm5*^*A7*^ mutants exhibit structurally normal SC, despite often experiencing heterosynapsis.

### Centromere-directed chromosome movements are normal in *Mcm5*^*A7*^ mutants

**Fig 3:**
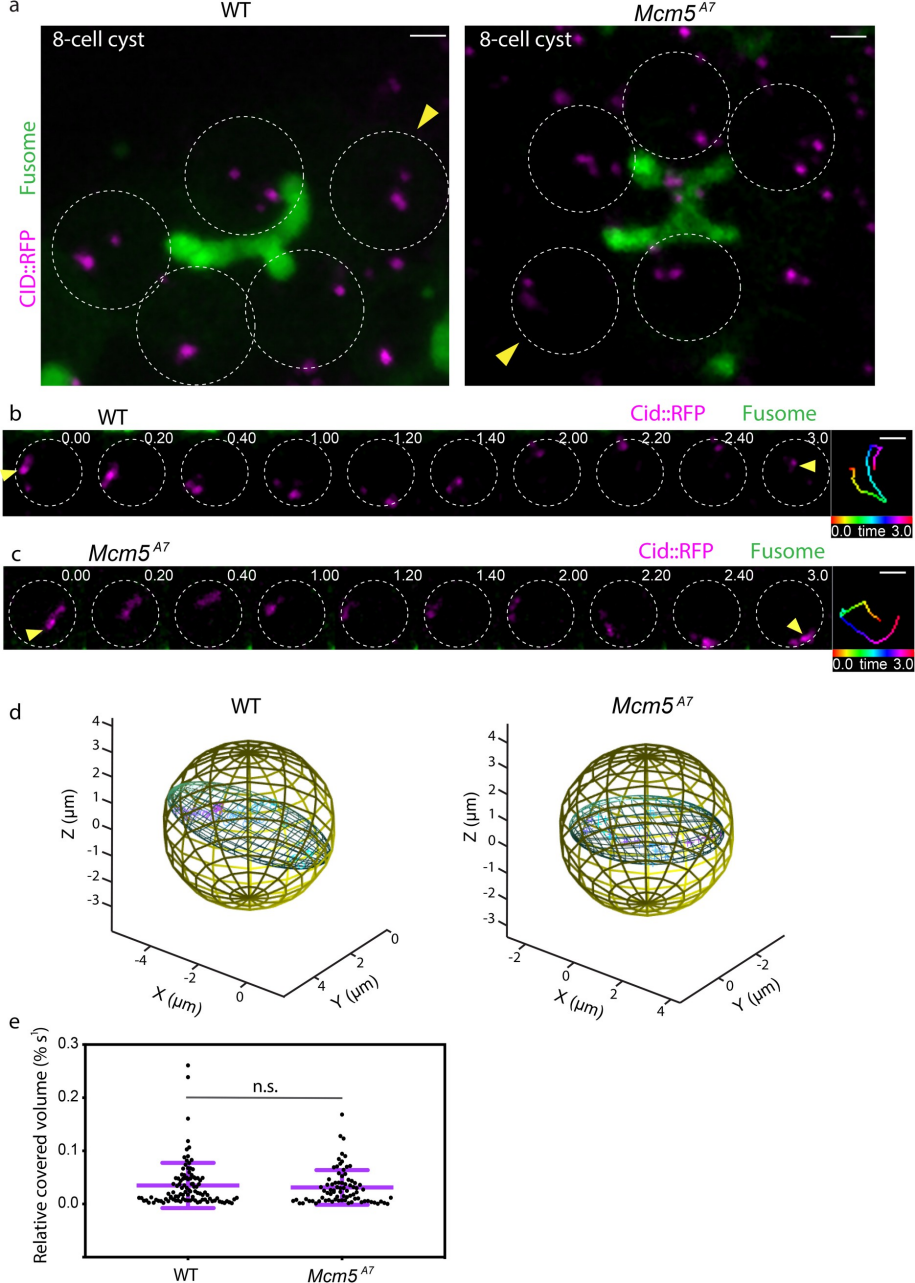
Centromeres in *Mcm5*^*A7*^ mutants exhibit dynamic, rapid movements. a. Projection of *Z*-sections of live *WT* (left) and *Mcm5*^*A7*^ 8-cell cysts expressing CID::RFP (magenta) and Par-1::GFP (fusome, green). Circles represent individual nuclei within the 8-cell cysts. Yellow arrow heads denote representative analyses shown in b. and c. and quantified by time points in d. Scale bars = 2μm. For videos, refer to S3 and S4 Movies. b. Selected projections from one *WT* 8-cell cyst nucleus in (A, indicated by yellow arrow head) over a 3-minute time course. See S5 Movie for full movie. c. Selected projections from one *Mcm5*^*A7*^ 8-cell cyst nucleus in (A, indicated by yellow arrow head) over a 3-minute time course. See S6 Movie for full movie. Time-colored tracking for CID-RFP dots indicated by yellow arrow heads are shown in right panels for b. and c. Scale bars = 2μm. d. 3-dimensional representations demonstrating the covered volume of a representative track for all time points in *WT* (50 time points, volume = 12.9 μm^3^) and *Mcm5*^*A7*^ (48 time points, volume = 15.7 μm^3^). e. Distribution of the relative covered volume (raw covered volume/nuclear volume) per second for each track in *WT* (*n* = 103 centromere foci) and *Mcm5*^*A7*^ (*n* = 80 centromere foci). *p* = 0.75, Kolmogorov-Smirnov test. Data are represented as mean ± SD.

We set out to understand how initial pairing of homologs is proficient in *Mcm5*^*A7*^ mutants, despite exhibiting defects in pairing maintenance. In many species, rapid chromosome movements are thought to contribute to homolog pairing [reviewed in ref. 4]; in *Drosophila*, these movements are centromere-directed [7]. To determine whether perturbations in centromere-directed chromosome movement contribute to the pairing maintenance defects in *Mcm5*^*A7*^ mutants, we examined centromere dynamics in 8-cell cysts of wild-type and *Mcm5*^*A7*^ mutant germaria through live cell imaging (Fig 3A, S3 Movie, S4 Movie).

In wild type, a representative centromere track illustrates chromosome movement around the volume of a nucleus (Fig 3B), covering a nuclear volume of 12.9 μm^3^ (Fig 3D, S5 Movie). A representative centromere track in *Mcm5*^*A7*^ mutants shows similar chromosome movement (Fig 3C), covering a nuclear volume of 15.7 μm^3^ (Fig 3D, S6 Movie). In total, *Mcm5*^*A7*^ mutants show no significant difference between relative nuclear volume covered compared to wild-type (Fig 3E, *p* = 0.75, Kolmogorov-Smirnov test), demonstrating that *Mcm5*^*A7*^ mutants exhibit centromere-directed chromosome movements similar to wild-type in the 8-cell cyst. Importantly, these data show that centromere-directed chromosome movement may promote initial meiotic homolog pairing but is not sufficient for maintaining homolog pairing.

### Meiotic centromere clustering is defective in *Mcm5*^*A7*^ mutants

**Fig 4:**
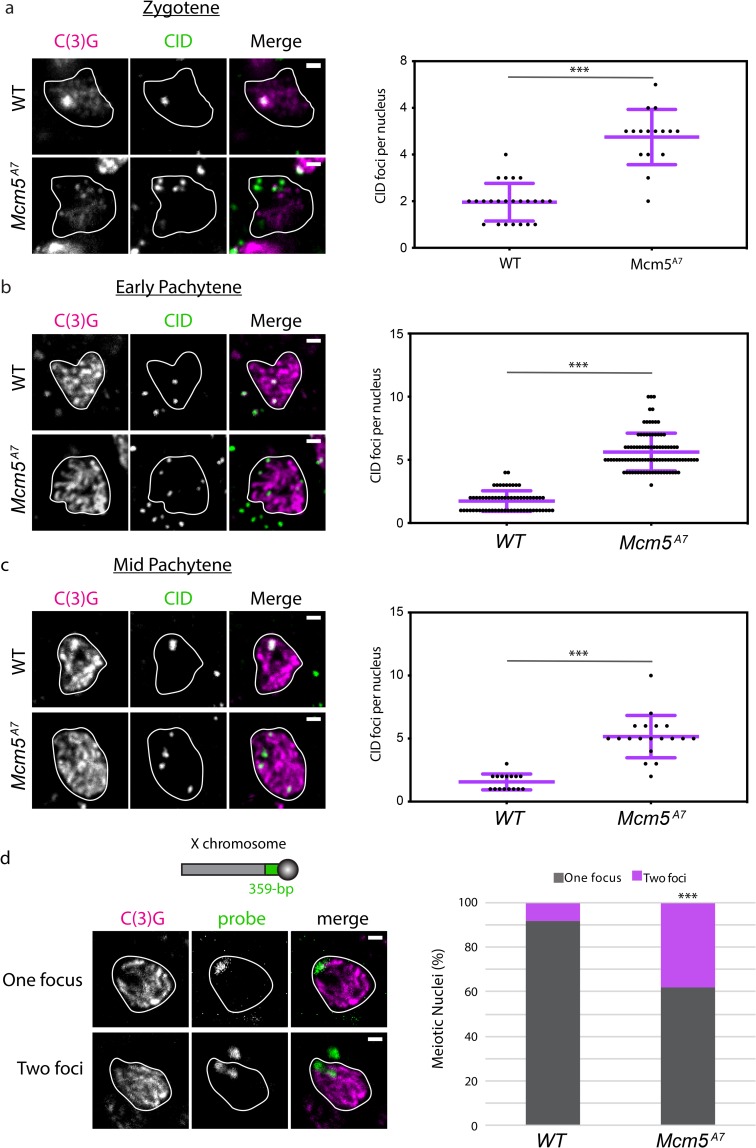
Centromere clustering is disrupted in *Mcm5*^*A7*^ mutants. a. Left: Representative images of centromere clustering, or lack thereof, in wild-type (*WT*) and *Mcm5*^*A7*^ meiotic nuclei located in zygotene. Magenta: C(3)G, green: CID (centromere). In these images, *WT* nucleus contains 1 CID focus, and *Mcm5*^*A7*^ contains 6 CID foci. Scale bar = 1 μm. Right: Quantification of CID foci in zygotene in *WT* (*n* = 24) and *Mcm5*^*A7*^ (*n* = 16). ***p < 0.0001, unpaired T-test. Data are represented as mean ± SD. b. Left: Representative images of centromere clustering in *WT* and *Mcm5*^*A7*^ early pachytene nuclei. Magenta: C(3)G, green: CID. In these images, *WT* nucleus contains 2 CID foci, and *Mcm5*^*A7*^ contains 5 CID foci. Scale bar = 1 μm. Right: Quantification of early pachytene CID foci in *WT* (*n* = 65) and *Mcm5*^*A7*^ (*n* = 94). ***p < 0.0001, unpaired T-test. Data are represented as mean ± SD. c. Left: Representative images of centromere clustering, or lack thereof, in mid-pachytene *WT* and *Mcm5*^*A7*^ nuclei. Magenta: C(3)G, Green: CID. In these images, *WT* nucleus contains 1 CID focus, and *Mcm5*^*A7*^ contains 4 CID foci. Scale bar = 1 μm. Right: Quantification of mid-pachytene CID foci in *WT* (*n* = 16) and *Mcm5*^*A7*^ (*n* = 19). ***p < 0.0001, unpaired T-test. Data are represented as mean ± SD. d. Schematic representing relative location of 359-bp locus on Chromosome *X* (not drawn to scale). Top panel: Representative image of meiotic nucleus with 1 359-bp (green) focus (*WT*, Region 2A). Bottom panel: Representative image of meiotic nucleus with 2 359-bp (green) foci (*Mcm5*^*A7*^, Region 2A). Right: Percentage of nuclei with paired 359-bp loci (one focus) or unpaired (two loci) in *WT* (*n* = 88) and *Mcm5*^*A7*^ (*n* = 63) meiotic nuclei. ***p < 0.0001, as determined by two-tailed Fisher’s exact test. Contrast and brightness of all images were adjusted for clarity. Outlines of representative nuclei are drawn in white. CID foci not localized with C(3)G are from adjacent, non-meiotic cells. All DAPI-containing images are provided in S2 Fig.

In *Drosophila*, eight centromeres aggregate into one or two diffraction-limited clusters at the onset of meiosis, which is defined cytologically as zygotene. Meiotic centromeres remain clustered through pachytene, while nurse cells do not experience centromere clustering [11]. To determine whether centromere clustering at the onset of meiosis is associated with initial homolog pairing, we quantified the number of foci of CENP-A ortholog CID at zygotene (Fig 4A). We observed the expected one or two CID foci in most wild-type nuclei, demonstrating centromere clustering. In *Mcm5*^*A7*^ mutants, we see a significant increase in CID foci, with a mean of 4.8 per nucleus (*p* < 0.001, unpaired T-test). These results show that in *Mcm5*^*A7*^ mutants, centromeres are not heterologously clustered entering meiosis, even though chromosome arms are paired.

Defects in centromere clustering in *Mcm5*^*A7*^ mutants extend into pachytene. As shown in Fig 4B, we observe a mean of 1.7 CID foci in early pachytene nuclei of wild-type, compared to 5.6 foci in *Mcm5*^*A7*^ mutants (*p* < 0.001, unpaired T-test). In mid-pachytene, *Mcm5*^*A7*^ mutants exhibit a mean of 5.2 CID foci, significantly higher than wild-type (1.6 CID foci; *p* < 0.001, unpaired T-test) (Fig 4C). Interestingly, in some *Mcm5*^*A7*^ meiotic nuclei, we observed more than eight CID foci, suggesting that sister centromere cohesion may be compromised. We conclude that centromere clustering and possibly centromere cohesion are perturbed throughout early and mid-pachytene in *Mcm5*^*A7*^ mutants.

In the regions assessed, we observed more than four CID foci in most *Mcm5*^*A7*^ nuclei (Fig 4A, 4B and 4C), suggesting that homologous centromeres are unpaired. To assay this directly, we examined the frequency of pairing of the 359-bp locus, the repeat unit of the 1.688 satellite in the pericentromeric heterochromatin of the *X* [16, 17] (Fig 4D). As previously reported, this locus is paired in ~90% of wild-type pachytene cells [8]; however, in *Mcm5*^*A7*^ pachytene nuclei, 359-bp locus pairing is significantly reduced to 61% (*p* < 0.001, two-tailed Fisher’s exact test). We conclude that meiotic homologous centromere pairing and heterologous centromere clustering are severely decreased, if not eliminated, in *Mcm5*^*A7*^ mutants.

### SMC1 enrichment at the centromere is reduced in *Mcm5*^*A7*^ mutants

Centromere clustering is perturbed in some cohesin and SC mutants [9, 11, 12], suggesting that specific proteins at the centromeres are required for clustering. Since we see no decrease of C(3)G at centromeres in *Mcm5*^*A7*^ mutants compared to wild type (S1 Fig), we hypothesized that a defect in cohesin localization may contribute to the decrease in centromere clustering. We examined localization of the cohesin SMC1, which is common to two meiosis-specific cohesion complexes that localize at the centromere and the chromosome arm [18].

**Fig 5:**
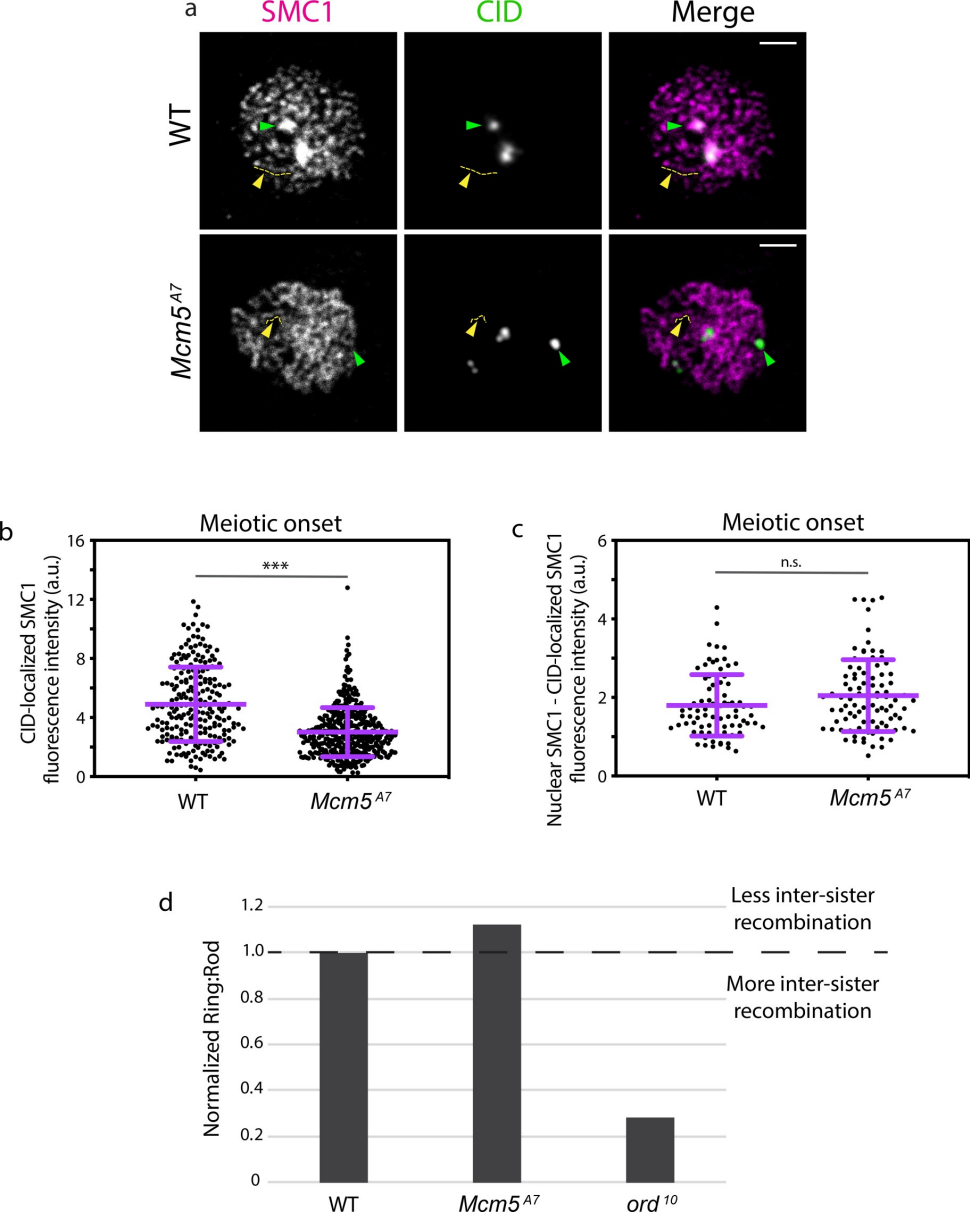
Centromeric SMC1 is significantly reduced in *Mcm5*^*A7*^ mutants. a. Representative images of chromosome spreads in *WT* and *Mcm5*^*A7*^ meiotic nuclei examining localization of SMC1 (magenta) and CID (green). Green arrow: SMC1 enrichment at the centromere, yellow arrow and tract: SMC1 along the chromosome arm. Scale bar = 2 μm. Contrast and brightness of images were adjusted for clarity. b. Integrated SMC1 fluorescence intensity at CID foci in meiotic nuclei at meiotic onset (zygotene + early pachytene, Region 2A) at *WT* (*n* = 225) and *Mcm5*^*A7*^ (*n* = 398) meiotic centromeres. ***p < 0.0001, unpaired T-test. Data are represented as mean ± SD. c. CID-localized SMC1 fluorescence intensity subtracted from total nuclear SMC1 fluorescence in meiotic nuclei at meiotic onset (zygotene + early pachytene, Region 2A) in *WT* (*n* = 81) and *Mcm5*^*A7*^ (*n* = 93) meiotic nuclei. *n*.*s*. = 0.0548, unpaired T-test. Data are represented as mean ± SD. S3A Fig for representative images. d. *Mcm5*^*A7*^ (*n* = 1194) and *ord*^*10*^ (*n* = 250) mutants examined for inter-sister recombination through the ratio of Ring chromosome to Rod chromosome transmission. *WT* (*n* = 2574 for *Mcm5*^*A7*^ experiment, *n* = 1204 for *Ord* experiment) was normalized to 1. Ratios above 1 suggest less inter-sister recombination; ratios below 1 suggest more inter-sister recombination. Refer to S1 Table for complete dataset. For further details about the assay, see S3B–S3D Fig.

At meiotic centromeres, SMC1 is a component of a complex that also contains SMC3 and the meiosis-specific proteins SUNN, SOLO, and ORD [18]. Centromeric enrichment of SMC1 is apparent in chromosome spreads [10] of wild-type meiotic nuclei (Fig 5A, green arrowhead). In *Mcm5*^*A7*^ mutants, however, centromeric enrichment of SMC1 appears to be reduced or absent. We quantified SMC1 localization in nuclei from whole mount germaria at meiotic onset (defined cytologically as zygotene and early pachytene, which cannot be distinguished based on SMC1 patterning). At meiotic onset, SMC1 fluorescence at the centromere is significantly reduced in *Mcm5*^*A7*^ mutants compared to wild type (Fig 5B, ***p < 0.001).

The cohesin complex described above also localizes along the chromosome arm with a second cohesin-like complex; this second complexes consists of SMC1 and SMC3, as well as C(2)M and SA [18]. Together, these two complexes comprise the axial element that forms between the arms of sister chromatids and later serves at the lateral element of the SC (Fig 2A). The ribbon-like patterning of SMC1 (Fig 5A, yellow arrowhead and line) represents the axial element that consists of the two SMC1-containing complexes. In chromosome spreads, SMC1 localization along the arms appears similar between wild type and *Mcm5*^*A7*^ (Fig 5A). When centromere-localized SMC1 fluorescence is subtracted from total nuclear SMC1 fluorescence of whole-mount germaria, we observe no significant difference between wild-type and *Mcm5*^*A7*^ mutants (*p* = 0.0548, Fig 5C). These observations suggest that SMC1 localization on chromosome arms is unaffected by the *Mcm5*^*A7*^ mutation.

Cohesion function along the arms also appears to be intact in *Mcm5*^*A7*^ mutants. In our FISH experiments, we never observed more than two foci, as would be expected if there were arm cohesion defects [19] (Fig 1B and 1C). An important function of the axial element is to provide a barrier to recombination between sister chromatids [20–22]. We examined inter-sister recombination rates using a genetic ring/rod chromosomal transmission assay (Figs 5D and S3B–S3D). An axial element mutant (*ord*^*10*^) shows a severe decrease in ring:rod transmission (normalized ratio of 0.28). In contrast, *Mcm5*^*A7*^ mutants exhibit no decrease in ring:rod transmission (1.1 ratio) [23], consistent with a functional axial element.

### Increasing centromeric SMC1 ameliorates pairing defects

Using *Mcm5*^*A7*^ mutants, we observed that a decrease in centromeric SMC1 localization during meiotic onset is associated with a reduction in meiotic centromere clustering and homologous chromosome pairing in pachytene, but not chromosome pairing in zygotene. Thus, we hypothesized that the centromeric SMC1 defect at meiotic onset causes the reduction in centromere clustering and the defect in pairing maintenance.

**Fig 6:**
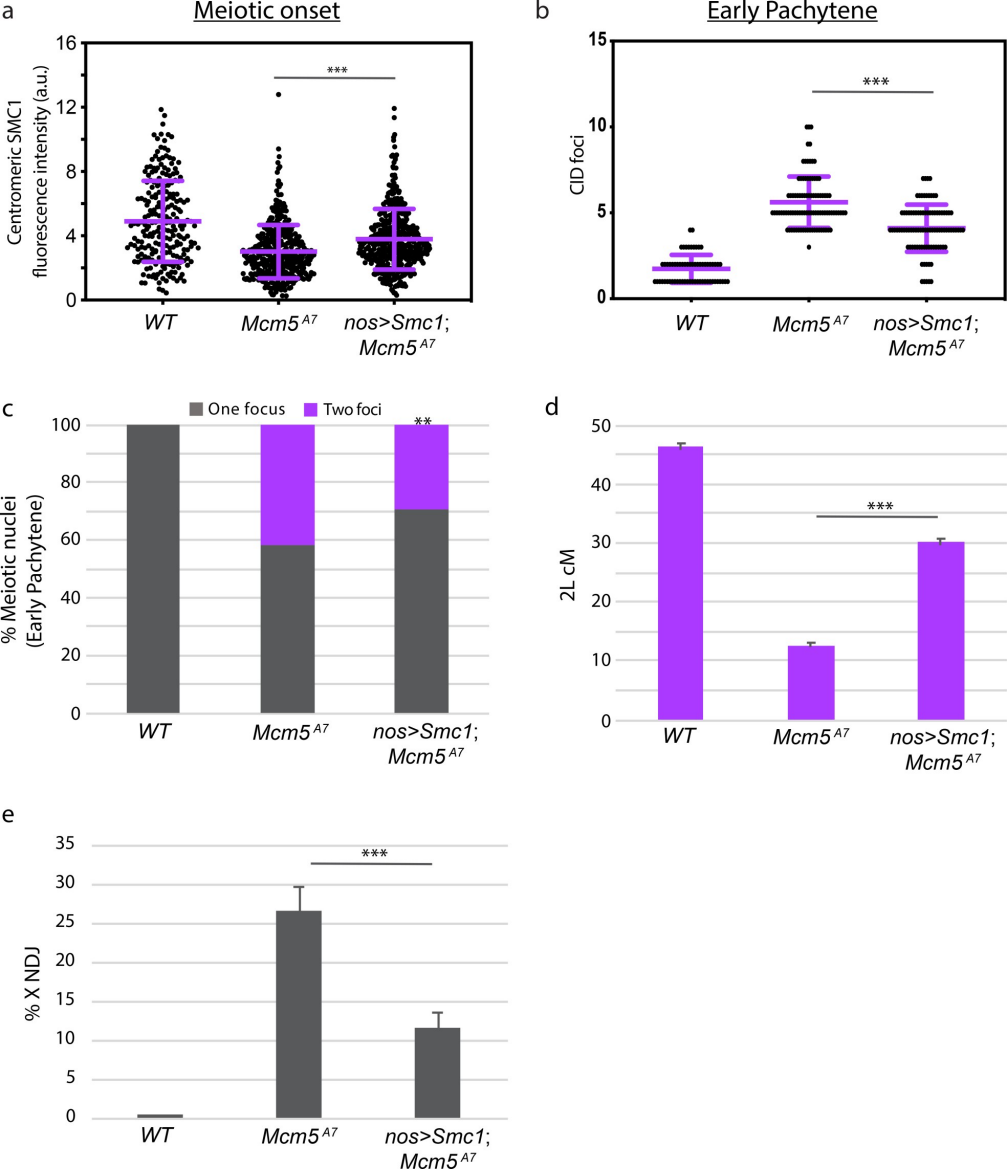
Overexpression of SMC1 in *Mcm5*^*A7*^ mutants rescue clustering, pairing, crossover formation, and NDJ. a. Integrated SMC1 fluorescence intensity at *WT*, *Mcm5*^*A7*^, and *nos>Smc1; Mcm5*^*A7*^ (*n* = 427) meiotic centromeres at meiotic onset. See S4A Fig for representative images of *nos>Smc1; Mcm5*^*A7*^ nuclei. *WT* and *Mcm5*^*A7*^ data are repeated from Fig 5. ***p < 0.0001, unpaired T-test. Data are represented as mean ± SD. b. Number of centromeres (CID foci) in *WT*, *Mcm5*^*A7*^, and *nos>Smc1; Mcm5*^*A7*^ (*n* = 94) meiotic nuclei at early pachytene. *WT* and *Mcm5*^*A7*^ data are repeated from Fig 2. ***p < 0.0001, unpaired T-test. Data are represented as mean ± SD. c. Percent of total paired and unpaired in *WT*, *Mcm5*^*A7*^, and *nos>Smc1; Mcm5*^*A7*^ (total *n* = 169) nuclei at early pachytene, combining *X*-probe and *3*R-probe data. *WT* and *Mcm5*^*A7*^ data are repeated from Fig 4 and are represented as *X*-probe plus *3*R-probe early pachytene data. Significance comparing *Mcm5*^*A7*^ and *nos>Smc1*, *Mcm5*^*A7*^: **p = 0.0002, chi-square d. Crossover levels on chromosome *2*L as shown in cM in *WT* (*n* = 4222) [54], *Mcm5*^*A7*^ (*n* = 2070), and *nos>Smc1; Mcm5*^*A7*^ (*n* = 933). ***p < 0.001, chi-square. Data are represented as mean ± 95% CI. Refer to S2 Table for full *2*L crossover dataset. e. NDJ of the *X* chromosome in *WT* (0.07%, *n* = 3034), *Mcm5*^*A7*^ (26.5%, *n* = 1979), *nos>Smc1; Mcm5*^*A7*^ (11.5%, *n* = 2282). ***p < 0.0001 as calculated by [48]. Data are represented as mean ± 95% CI. Refer to S3 Table for full NDJ dataset.

To test this hypothesis, we attempted to restore SMC1 localization at the meiotic centromere in *Mcm5*^*A7*^ mutants through exogenous expression [18]. Expression of an individual cohesion complex subunit may increase expression of other subunits, on both transcriptional and translational levels, possibly resulting in a functional complex [24, 25]. Using quantitative microscopy, we found that centromeric SMC1 is significantly higher in *nos>Smc1; Mcm5*^*A7*^ than in *Mcm5*^*A7*^ mutants at meiotic onset (***p < 0.0001, unpaired T-test) (Fig 6A). We next assayed centromere clustering at early pachytene, when we first observe pairing defects in *Mcm5*^*A7*^ mutants (Fig 1B); as shown in Fig 6B, centromere clustering was significantly increased in *nos>Smc1; Mcm5*^*A7*^ as compared to *Mcm5*^*A7*^ (***p < 0.0001, unpaired T-test), indicating that the increase in centromeric SMC1 localization at meiotic onset partially rescues the early pachytene centromere clustering deficiency in *Mcm5*^*A7*^ mutants. Next, we examined pairing frequency of *X* and *3*R at early pachytene in *nos>Smc1; Mcm5*^*A7*^ flies (Fig 6C). We see a significant pairing increase in *nos>Smc1; Mcm5*^*A7*^ mutants compared to *Mcm5*^*A7*^ mutants (pairing frequency of 71% and 59%, respectively, **p = 0.0066, chi-square). From these data, we conclude that the defect in centromeric SMC1 localization in *Mcm5*^*A7*^ mutants causes the observed defects in centromere clustering and meiotic pairing stability.

We initially hypothesized that a lack of homolog pairing results in the loss of meiotic crossovers in *Mcm5*^*A7*^ mutants. We reasoned that in the presence of heterosynapsis, as seen in *Mcm5*^*A7*^ mutants, meiotic DSBs cannot be repaired into crossovers because no homologous template is available. To test this hypothesis, we measured crossovers across chromosome *2L* in wild-type, *Mcm5*^*A7*^, and *nos>Smc1; Mcm5*^*A7*^ mutants (Fig 6D). In wild-type flies the genetic length of this region is 45.8 cM, but in *Mcm5*^*A7*^ mutants this is reduced to 12.3 cM (***p < 0.0001, chi-square). In *nos>Smc1; Mcm5*^*A7*^ mutants there is significant rescue of this defect, to 29.8 cM (*** *p* < 0.0001, chi-square, as compared to *Mcm5*^*A7*^ mutants). These results indicate that the pairing defect and heterosynapsis during early pachytene is, at least in part, the cause for the loss of crossovers in *Mcm5*^*A7*^ mutants.

Because crossovers are partially rescued in *nos>Smc1; Mcm5*^*A7*^ flies, the high nondisjunction rate in *Mcm5*^*A7*^ should be lessened when SMC1 is overexpressed in these mutants. We observe that *nos>Smc1; Mcm5*^*A7*^ mutants have a significant decrease in *X-*NDJ as compared to *Mcm5*^*A7*^ mutants (NDJ rate of 11.5% and 26.5%, respectively, Fig 6E) (***p<0.0001). Thus, germline overexpression of SMC1 can restore SMC1 at the centromere in *Mcm5*^*A7*^ mutants in early pachytene, leading to improved centromere clustering, homolog pairing and synapsis, crossover formation, and chromosome segregation.

## Discussion

**Fig 7:**
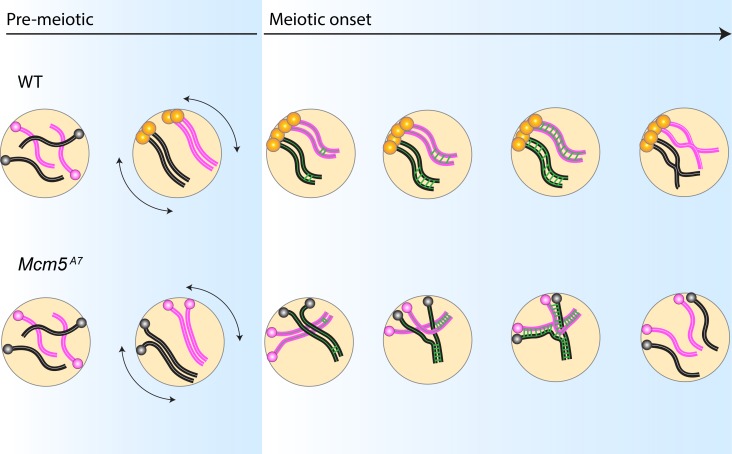
Centromeric SMC1-dependent pairing model. *WT*: In pre-meiotic cysts, homologous chromosomes (pink = homolog pair 1, black = homolog pair 2) enter the germline unpaired. During pre-meiotic cell cycles, chromosome arms and centromeres pair, with centromeres anchored at the nuclear envelope. Prior to meiotic onset, SMC1 is enriched at the centromeres (yellow) and centromere-directed chromosome movement (double-headed arrows) occurs. These events yield centromere clustering at meiotic initiation. As euchromatic synapsis nucleates along arms (green bars), paired chromosomes are stabilized by centromere clustering, permitting homosynapsis. After DSB formation and repair (not depicted), crossovers between homologs are formed, promoting proper disjunction at the end of meiosis I. *Mcm5*^*A7*^: Chromosomes enter the germline unpaired, and centromeres are attached to the nuclear envelope. In pre-meiotic cycles, chromosomes initially pair, but centromeres do not. Centromere-directed chromosome movements occur, but SMC1 is not enriched at the centromere, causing a lack of centromere clustering at meiotic onset. As the SC nucleates at the arms, homolog pairing is not maintained. Synapsis spreads between the nearest chromosomal regions, independent of homology, yielding high frequency of heterosynapsis. During heterosynapsis, DSBs are not repaired by HR, yielding non-recombinant chromosomes that nondisjoin at the end of Meiosis I.

For successful meiosis, homologs must pair, and this pairing must be maintained during synapsis; these processes are both largely a mystery. At the beginning of this study, we hypothesized that the crossover defect in *Mcm5*^*A7*^ mutants was due to a homolog pairing deficiency. Our FISH results support this hypothesis (Fig 1) and revealed that homolog pairing can be lost during synapsis, resulting in seemingly normal SC between heterologous sequences (Fig 2). Centromere-directed chromosome movements occur in *Mcm5*^*A7*^ mutants (Fig 3), presumably to promote initial chromosome arm pairing; however, centromere pairing and clustering are perturbed (Fig 4). SMC1 enrichment at the centromere is decreased in *Mcm5*^*A7*^ mutants (Fig 5), while arm cohesion appears normal. Overexpression of SMC1 rescues centromeric-SMC1 localization and downstream meiotic defects, including centromere clustering, pairing, crossover formation, and segregation (Fig 6). From our data, we propose that centromeric SMC1 stabilizes initial homolog pairing through centromere clustering, securing meiotic pairing, ensuring homosynapsis and promoting crossover formation (Fig 7).

### SMC1-dependent centromere clustering homolog pairing stability model

Prior to the onset of meiosis, cohesins are loaded onto centromeres [10], and homologous chromosomes pair, with arm pairing preceding centromere pairing [8, 9]. Initial homolog pairing is achieved in part by centromere-directed movements in the division prior to meiotic onset [7]. We propose a model in which initial homologous chromosomal pairing is stabilized throughout early meiosis by SMC1-dependent centromere clustering (Fig 7).

According to this model, the enrichment of SMC1 at the centromere combined with chromosome movements in pre-meiotic stages yield centromere clustering at meiotic onset. While chromosome arms and centromeres enter meiosis paired, heterologous centromere clustering and/or centromere pairing are required to stabilize pairing during SC assembly. As initial euchromatic SC patches extend along the arms of paired homologs, DSBs are formed and subsequently repaired via HR to yield crossovers, which promote accurate disjunction at the end of meiosis.

In *Mcm5*^*A7*^ mutants, coordinated pre-meiotic centromere-directed movements occur, but a sufficient amount of SMC1 is not localized at the centromere to yield centromere clustering. Thus, at meiotic onset, arms are paired, but centromeres are not clustered. As euchromatic SC nucleation occurs, the stabilization provided by centromere clustering is absent, and homologous loci become unpaired. As synapsis extends, the SC is able to form between nearby chromosomes, regardless of homology, yielding heterosynapsis (intrachromosomal and/or interchromomosomal). DSBs made within regions of heterosynapsis are not repaired via HR due to the absence of an available homologous template. Therefore, crossovers are reduced, and nondisjunction occurs at high frequency in *Mcm5*^*A7*^ mutants.

The SMC1-dependent centromere clustering pairing model highlights the finding that initial meiotic pairing is not sufficient to yield complete homosynapsis. Rather, centromeric SMC1-dependent stabilization must occur after pairing and during synapsis. The inherent requirement of pairing stabilization for proper synapsis suggests that there is a force that opposes homolog alignment during synapsis. Perhaps the SC assembly process itself creates an opposing force that can push paired homologs away from one another in the absence of stabilization; a similar hypothesis was previously proposed in *C*. *elegans* [26]. An alternative hypothesis is that recombination, which coincides temporally with synapsis assembly, creates a destabilizing force. However, when meiotic DSBs are eliminated in *Mcm5*^*A7*^ mutants (as shown through *mei-P22 Mcm5*^*A7*^ double mutants), homologs are unpaired at a frequency similar to *Mcm5*^*A7*^ mutants (S5 Fig), indicating that the opposing force is independent of recombination. Regardless of the origin of the force, we propose that SMC1-dependent centromere clusters act as anchors at the nuclear envelope to maintain the proximity of homologous axes.

Although meiotic pairing programs vary among organisms, the SMC1-dependent centromere clustering pairing model may be broadly applicable. In *Drosophila* and *C*. *elegans*, meiotic pairing is independent of meiotic recombination. In contrast, meiotic pairing in organisms such as yeast, plants, and mice require DSB formation (although recombination-independent alignment is required for pairing in these organisms) [2]. In DSB-dependent pairing programs, homologs are considered paired at ~400 nm, where DSB-mediated interhomolog interactions can be visualized as bridges [27]. However, contemporaneous with DSB formation, centromeres are coupled or clustered [reviewed in 28]. We speculate that these centromere interactions stabilize the DSB-dependent arm pairing to ensure synapsis exclusively between homologs in many sexually-reproducing organisms.

### Pairing and synapsis in *Drosophila*

This study reveals the interesting phenomenon of stable heterosynapsis in *Drosophila*. Extensive heterosynapsis has been previously reported in *C*. *elegans* [29–35] and yeast [36] with variable SC integrity. Though we cannot rule out SC aberrations in *Mcm5*^*A7*^ mutants, our data reveal no structural defects, supporting the notion that “normal” synapsis is largely homology-independent in *Drosophila*, as has been observed in *C*. *elegans* [37].

In *Drosophila*, synapsis along the arms initiates as patches during zygotene [12]. In *Mcm5*^*A7*^ mutants, synapsis initiation between paired homologs appears normal in zygotene but SC elongation fails to be limited to homologous regions. Thus, initiation of synapsis may require homology, but elongation may not. Because our study examined only specific loci and not whole chromosomes, future studies determining the degree of heterosynapsis in *Mcm5*^*A7*^ mutants may provide more insight into how synapsis and homology interact in flies.

### Effects of the *Mcm5*^*A7*^ mutation

While *Mcm5*^*A7*^ has proven to be a valuable genetic tool, how this particular mutation affects SMC1 localization at the meiotic centromere is unknown. *Mcm5*^*A7*^ mutants do not affect centromere clustering and pairing in a manner similar to that of mutants that disrupt centromere integrity, such as *cal1 Cenp-C* double heterozygotes (S5 Fig) [38]. However, our results do not exclude a role for MCM5 in centromere function or integrity.

The canonical role of MCM5 is to function within the replicative helicase, MCM2-7, unwinding double-stranded DNA ahead of the replication fork during S-phase [reviewed in 39, 40]. Because of its important replication role, *Mcm5* is an essential gene in every proliferating cell. Numerous studies have shown that replication is required for cohesion localization and establishment [reviewed in 41], but examining a direct role for any MCM protein in cohesin deposition is difficult since MCMs are essential for replication, which in turn is required for establishing cohesion.

Because MCM5 functions within the MCM2-7 replicative helicase, it is tempting to speculate that the *Mcm5*^*A7*^ mutation may directly perturb SMC1 localization, either through defects in replication or cohesin deposition. No replication defect in *Mcm5*^*A7*^ mutants has been detected, in either a mitotic or meiotic context [1]. In the future, when individual pre-meiotic nuclei can be isolated from cysts, higher-resolution replication assays may determine whether replication is subtly disrupted in *Mcm5*^*A7*^ mutants. At this point, however, it seems more likely that the *Mcm5*^*A7*^ cleanly separates the replication role of MCM5 from a role in meiotic cohesin deposition.

## Methods

### Experimental model details

In all experiments, *Drosophila melanogaster* adult females 3–10 days old were used. Flies were maintained on standard medium at 25°C. *Drosophila* nomenclature used in this study was generalized for readership. Nomenclature and specific genotypes are listed below (Table 1).

**Table 1:** Genotypes used in this study.

Manuscript Nomenclature	*Drosophila* genotype(s)	Figure(s)
*Mcm5*^*A7*^	*Mcm5*^*A7*^ [1]	1, 2, 4, 5, 6, S1, S2, S3, S4
	*Df(3R)Exel7305* [42]	
*WT*	*y w*^*1118*^	1, 2, 4, 5, 6, S1, S2, S3, S4
*ord*^*10*^	*ord*^*10*^ [21]	2
*WT CID*::*RFP*	*w; P{nanos*::*GAL4}; CID*::*RFP* [7]	3
	*w; P{UAS*::*Par1-GFP}* [7]*; +*	
*Mcm5*^*A7*^ *CID*::*RFP*	*w; P{nanos*::*GAL4}; Mcm5*^*A7*^, *CID*::*RFP*	3
	*w; P{UAS*::*Par1-GFP}; Df(3R)Exel7305*	
*nos>Smc1; Mcm5*^*A7*^	*w; P{nanos*::*GAL4}; Df(3R)Exel7305*	6, S4
	*w; P{UAS*::*Smc1-HA}; Mcm5*^*A7*^	
*nos>Smc1; Mcm5*^*D/+f*^	*w; P{nanos*::*GAL4}; Df(3R)Exel7305*	S4
	*y w*, *P{UAS*::*Smc1-HA}* [18]*; +*	
*Mcm5>Mcm5*^*WT*^*; Mcm5*^*A7*^	*w; P{Mcm5*::*Mcm5}* [1]*; Mcm5*^*A7*^	S4
	*+; Df(3R)Exel7305*	
*rec*^*1/2*^	*rec*^*1*^ [43]	S4
	*rec*^*2*^ [44]	
*nos>Smc1; rec*^*1/2*^	*w; P{nanos*::*Gal4}; rec*^*1*^	S4
	*y w*, *P{UAS*::*Smc1-HA}; rec*^*2*^	
*cal1*^*2K32*^ + / + *cenp-c*^*pr141*^	*Cenp-C*^*pr141*^ [45]	S5
	*cal1*^*2K32*^ [46]	S5
*mei-P22*	*mei-P22*^*103*^ [47]	S5

### Genetic assays

*X* chromosome NDJ was evaluated by scoring the progeny from virgin females of desired genotype crossed with *y cv v f / T(1*:*Y)B*^*S*^ males. Viable exceptional *XXY* females have *Bar* eyes, and viable exceptional *X*0 males have *Bar*^*+*^ eyes and are *y cv v f*. To adjust for inviable exceptional males and females, viable exceptional class was multiplied by 2. % *X*-NDJ = 100* ([2*viable exceptional females] + [2*viable exceptional males])/total progeny. Statistical comparisons were performed as in [48].

Crossovers on chromosome *2*L were measured by crossing virgin *net dpp*^*ho*^ *dp b pr cn /* + females of desired genotype to *net dpp*^*ho*^ *dp b pr cn* males. Vials of flies were flipped after three days of mating. Resulting progeny were scored for all phenotypic markers. Similarly, crossovers on chromosome *X* were measured by crossing virgin *y sc cv v g f y*^*+*^ */* + females to *y sc cv v g f* males. Progeny were assessed for all phenotypic markers.

To calculate intersister recombination, *R(1)2*, *y*^*1*^ *w*^*hd80k17*^ *f*^*1*^*/ y*^*1*^ females with desired genotype were crossed to *y*^*1*^ *w*^*1118*^ and progeny was scored for phenotypic markers. Exceptional progeny (progeny resulting from meiotic nondisjunction) were subtracted from total progeny and rates were adjusted to reflect only normal (non-exceptional) progeny, as in [21]. For complete dataset and calculation details, refer to S1 Table.

### Dissection and immunofluorescence (IF) of whole mount germaria

Ten three- to five-day old virgin females of desired genotype were fattened overnight with yeast paste in vials with ~5 males of any genotype. Ovaries were dissected in fresh 1x PBS and incubated in fixative buffer for 20 minutes. Fixative buffer: 165 μL of fresh 1x PBS, 10 μL of N-P40, 600 μL of heptane, and 25 μL of 16% formaldehyde. After being washed 3 times in 1x PBS + 0.1% Tween-20 (PBST), ovaries were incubated for 1 hour in 1 mL PBST + 1% BSA (10 mL of PBST + 0.1 g BSA). Ovaries were incubated overnight in 500 μL primary antibody diluted in 1 mL PBST + 1% BSA at 4° C on rocking nutator. After being washed 3x in PBST, ovaries were incubated in 500 μL secondary antibody diluted at 1:500 in PBST + 1% BSA for 2 hours under foil. Ovaries were mounted in 35 μL of ProLong Gold + DAPI on microscope slide using fine-tip forceps to spread ovaries.

Antibodies for C(3)G [49], SMC1[10], and CID (Active Motif) were used. For Fig 4A–4C, S2A–S2C Fig: Images of whole mount germaria were taken Zeiss LSM880 confocal laser scanning microscope using 63x/0.65 NA oil immersion objective, with a 2x zoom using ZEN software. Images were saved as .czi files and processed using FIJI [50]. For S1A and S1D Fig, S3A Fig, S4A Fig: Images were taken on Nikon A1R point-scanning confocal microscope using 60x/1.49 NA oil immersion objective. Images were saved as .nd2 files and quantified as described below.

### Dissection and IF of chromosome spreads

Before dissection, 25 mL of fixative, 5 mL of hypo-extraction buffer, and 500 μL of 100 mM sucrose were prepared. Fixative (25 mL): 23.0875 mL water, 1.5625 mL 16% formaldehyde, at 350 μL of 10% Triton-X (1 mL of Triton-X + 9 mL water). Hypo-extraction buffer (5 mL): 3.685 mL water, 250 μL 600 mM Tris (pH 8.2), 500 μL 170 mM Trisodium Citrate Dihydrate, 50 μL 500 mM EDTA, 2.5 μL 1.0 M DTT, 12.5 μL 200 mM Pefabloc (hypo-extraction buffer is good for only 2 hours). 100 mM Sucrose (500 μL): 100 μL 500 mM sucrose + 400 μL water).

Ovaries were dissected in 1x PBS and rinsed once in hypo-extraction buffer. Ovaries were incubated for 20 minutes in hypoextraction buffer and transferred to sucrose and minced. A super-frost slide was dipped into the fixative for 15 seconds. 10 μL of minced ovary tips were transferred onto the middle edge of the long side of the slide and rolled to allow spreading. Slides were dried very slowly overnight in a closed humidified chamber. Once dried, slides were incubated with 500 μL of blocking (5% normal goat serum (NGS), 2% BSA, 0.1% Triton-X in 1x PBS). Slides were rinsed 3 times in B-PBSTx (0.1% BSA, 0.1% Triton-X in 1x PBS). 250 μL of primary antibodies diluted in B-PBSTx were incubated under parafilm overnight in humidifying chamber. Slides were washed 3 times with PBSTx (0.1% Triton-X in 1x PBS). Secondary antibodies were diluted at 1:400 in B-PBSTx. 100 μL of diluted secondary were added onto slide under parafilm and incubated for an hour. Slides were rinsed 3 times in PBSTx and washed three time for 10 minutes in PBSTx in Coplin jar. Slides were incubated swith 400 μL DAPI (1 ug/ml) in 1x PBS for 10 minutes in dark and washed in 1x PBS. Coverslips were mounted with ProLong Gold.

Antibodies for C(3)G [49], Corolla [14], SMC1 [10], and CID (Active Motif) were used. Fig 5A: Images were taken on Zeiss LSM880 confocal laser scanning microscope using 63x/0.65 NA oil immersion objective with a 2x zoom using ZEN software. Images were saved as .czi files and processed using FIJI [50]. Fig 2D: Images were taken on Nikon N-SIM using Elements software. Images were saved as .nd2 files and processed using FIJI [50].

### Generation of fluorescence in situ hybridization (FISH) probes

DNA from desired BAC clones (BACPAC RPCI-98 Library) was extracted from MIDI-prep culture. For *X* probe (Fig 1B), six BAC clones were used, spanning cytological bands 6E-7B. Clones: 17C09, 06J12, 35J16, 20K01, 35A18, and 26L11. For *3*R probe (Fig 1C), six BAC clones were used, spanning cytological bands 93A-93E. Clones: 19 P12, 05 I01, 20 N14, 10 M16, 06 L13, 34 E13. The BAC-derived template DNA was used in a nick-translation reaction to generate euchromatic biotinylated DNA probes, as described below.

For one BAC clone DNA template, the following was added into a 0.5 mL tube: 5 μL 10X DNA Pol I buffer, 2.5 μL dNTP mix (1 mM each of dCTP, dATP, dGTP), 2.5 μL biotin-11-dUTP (1 mM), 5.0 μL 100 mM BME, 10 μL of freshly diluted dDNase I, 1 μL DNA Pol I, 1 ug of template DNA, water up to 50 μL. Reaction was incubated at 15° C in thermocycler for 4 hours and eluted in 20 μL TE. Concentration was determined using Qubit kit and diluted to a final concentration at 2 ng/μL in hybridization buffer. Hybridization buffer: 2x Saline-Sodium Citrate (SSC) buffer, 50% formamide, 10% w/v dextran sulfate, 0.8 mg/mL salmon sperm DNA.

The 359-bp probe (Fig 4D) was ordered from Integrative DNA Technologies (IDT, www.idtdna.com) with 5’ Cy5, resuspended in 1x TE at 100 μM. Sequence for 359-bp probe (5’ to 3’): Cy5- GGGATCGTTAGCACTGGTAATTAGCTGC.

### FISH/IF of whole mount germaria

Samples were prepped as described in [51]: Ovaries were dissected in fresh 1x PBS and incubated in fixative buffer for 4 minutes. Fixative buffer: 100 mM sodium cacodylate (pH 7.2), 100 mM sucrose, 40 mM potassium acetate, 10 mM sodium acetate, 10 mM EGTA, 5% formaldehyde. Ovaries were transferred to 0.5 ml tube filled with 2x SSCT (5 ml 20x SSC, 50 μL Tween, 45 mL water) and washed four times in 2x SSCT, 3 minutes each. Ovaries were washed 10 minutes in 2x SSCT + 20% formamide, 10 minutes 2x SSCT + 40% formamide, and then two times for 10 minutes each in 2x SSCT + 50% formamide. Ovaries were incubated at 37° C for 4 hours, at 92° C for 3 minutes and then 60° C for 20 minutes. Ovaries were transferred to tube with 36 μL of BAC-generated probe (diluted in hybridization buffer) or with 35 μL of hybridization buffer and 1 μL of IDT-generated probe. Ovaries were incubated in the thermocycler for 3 minutes at 91° C then at 37° C overnight and then washed with 2x SSCT + 50% formamide for 1 hour at 37° C. Ovaries were washed in 2x SSCT + 20% formamide for 10 minutes at room temperature and rinsed in 2x SSCT four times quickly. Ovaries were incubated for 4 hours in blocking solution (6 mg/mL NGS in 2x SSCT) and then washed three times quickly in 2x SSCT. Ovaries were incubated overnight in primary antibody diluted in 2x SSCT at room temperature. Ovaries were washed three times quickly in 2x SSCT and incubated for two hours in secondary antibody diluted in 2x SSCT. Biotinylated probes: sample was incubated in 1.5 μL of 488-conjugated streptavidin diluted in 98.5 μL detection solution (0.5 mL 1M Tris, 400 mg BSA, water to 10mL) for 1 hour, washed two times quickly in 2x SSCT, washed for 1 hour in 2x SSCT, and then washed 3 hours in 2x SSCT. (If using IDT-generated probes, these steps were not performed.) Ovarioles were mounted on a slide in 35 μL of DAPI + fluoromount.

Antibody for C(3)G [49] was used. Figs 1B and 1C and 4D: Images of whole mount germaria were taken on Zeiss LSM880 confocal laser scanning microscope using 63x/0.65 NA oil immersion objective with a 2x zoom using ZEN software. Fig 2B: Images were obtained using AIRY-Scan on Zeiss LSM880 confocal laser scanning microscope using 63x/0.65 NA oil immersion objective. Images were saved as .czi files and processed using FIJI [50].

### Live cell imaging

Ovaries were dissected in 10S Voltalef oil. The muscular sheath around each ovariole was removed and ovarioles were manually separated. Individual ovarioles were transferred to a drop of oil on coverslip. Videos were collected with an on an inverted Zeiss Axioobserver Z1 with motorized XYZ spinning-disc confocal microscope operated by Metamorph coupled to a sCMOS (Hamamatsuorca) camera and a temperature control chamber. All images were acquired with the Plan-Apochromat 100x/1.4 oil objective lens. Single-position videos in the germarium were acquired for 8 minutes at 25 ± 1° C, with a 10 second temporal resolution (12-slice Z-stack, 0.5 μm per slice).

### Recombination calculations

Genetic distances are expressed in centiMorgans (cM), calculated by 100 * (*R* / *n*), where *R* is the number of recombinant progeny in a given interval (including single, double, and triple crossovers), and *n* is the total number of progeny scored. 95% confident intervals were calculated from variance, as in Stevens [52]. Molecular distances (in Mb) are from the positions of genetic markers on the *Drosophila melanogaster* reference genome, release 6.12 [53]. Crossover density (or frequency), as calculated by cM/Mb, exclude transposable elements [51, 54, 55].

### Quantitative microscopy analysis of IF in whole mounts

For fixed germaria, DAPI and anti-CID with either anti-C(3)G or anti-SMC1 stains were used. Individual nuclei were first selected and eight 0.5 μm z-slices were used for analysis. Fluorescence intensities were measured following automated cytoplasmic background subtraction from individual slices. For centromeric fluorescence intensities, centromeres were first segmented based on anti-CID (Active Motif) fluorescence using a probabilistic segmentation approach. Using segmented centromere masks, integrated centromeric CID, C(3)G and SMC1 fluorescence intensities were quantified as sums across centromeres in individual nuclei. For nuclear fluorescence intensities, nuclei were segmented using anti-C(3)G fluorescence. To calculate chromosome arm fluorescence intensities, centromeric fluorescence intensities were subtracted from nuclear fluorescence intensities. To account for potential staining heterogeneity, fluorescence intensities were normalized to total nuclear CID fluorescence intensity, which was assumed to be unperturbed. Fluorescence intensities represent raw integrated densities of maximum intensity projected z-stacks. Nucleus selection, background subtraction and fluorescence intensity measurements were performed semi-automatically using custom FIJI-based plugins [50]. The custom FIJI-based plug-ins are publicly available at GitHub (https://github.com/viboud12/Germarium_Meiosis).

### Analysis of pairing and centromere clustering

To determine the meiotic stage in fixed whole mount germaria, nuclear C(3)G staining patterning was used. Spots of C(3)G in early Region 2A was considered zygotene, full-length C(3)G in Region 2A was considered early pachytene, and full-length C(3)G in Region 3 was considered mid-pachytene. Two foci were considered unpaired if distances between the center of the foci were equal to or greater than 0.7 um [56]. For centromere counting, any distinguishable single CID focus was counted as one, and distance between CID foci was not considered.

### Live cell imaging tracking

The use of PAR1::GFP on live germaria allowed the identification of the different cyst stages. For live germaria, images shown are the projection of all Z-series of a single (t) projection. Three-dimensional tracking of spinning-disc data was performed using Imaris software (Bitplane). The CID::RFP signal was tracked using the ‘spots’ function with an expected diameter of 0.3 μm. Automatically generated tracks were then edited manually to eliminate inappropriate connections, including connections between foci in different nuclei or between foci of different sizes or intensity when more likely assignments were apparent or multiple spots assigned to the same focus.

To remove global movements of the germarium, each nucleus containing a CID::RFP focus was assigned to the nearest fusome foci. Then, the position of the reference fusome was subtracted from each CID::RFP focus for each time point of the tracking to get the relative tracks. These relative tracks were then compiled using a custom MATLAB (MathWorks) routine that computes the minimum volume of the ellipsoid that encloses all of the three-dimensional points of the trajectory.

To analyze centromere trajectories: Positions of individual centromeres were tracked every 10 seconds for 8 minutes to quantify the volume covered by each centromere. This raw volume was then corrected both for overall movements of the tissue and for variations in total nuclear volume. First, we subtracted the motion of the germarium using the position of the fusome as a reference within each cyst. Second, to take into account the nuclear volume at 8cc, we computed the relative volume, which is the raw volume divided by the mean value of the nuclear volume at 8cc stage. Finally, we normalized durations of each track by calculating the relative covered volume per second (as shown in Fig 3E).

### Transparent reporting

Each microscopy experiment performed in this study was repeated independently at least two times. We did not use explicit power analysis; rather, in each experiment, at least 8 independent germaria were imaged, and meiotic cells within the germaria were quantified, giving the final sample size per experiment. The total number of samples (*n*) is the sum of the final sample sizes per experiment.

## Supporting information

S1 Fig C(3)G localization appears normal in *Mcm5*^*A7*^ mutants. a. Representative images of *WT* and *Mcm5*^*A7*^ meiotic nuclei in whole mount germaria that were quantified in b., c., and Fig 2E examining C(3)G (magenta) and CID (green) in early pachytene. b. Quantification of C(3)G signal at the centromere (CID) in *WT* and *Mcm5*^*A7*^ early pachytene nuclei. *p* = 0.4327, unpaired T-test. Data are represented as mean ± SD. c. Quantification of C(3)G signal at chromosome arm in *WT* and *Mcm5*^*A7*^ early pachytene nuclei. *p* = 0.6358, unpaired T-test. Data are represented as mean ± SD. d. Representative images of *WT* and *Mcm5*^*A7*^ meiotic nuclei of whole mount germaria that were quantified in e., f., and Fig 2F examining C(3)G (magenta) and CID (green) at mid-pachytene. e. Quantification of C(3)G signal at the centromere (CID) in *WT* and *Mcm5*^*A7*^ mid-pachytene nuclei. *p* = 0.3615, unpaired T-test. Data are represented as mean ± SD. f. Quantification of C(3)G signal at chromosome arms in *WT* and *Mcm5*^*A7*^ mid-pachytene nuclei. *p* = 0.5489, unpaired T-test. Data are represented as mean ± SD.
(TIF)

S2 Fig Multiple germarium nuclei are depicted per frame. (a, b, c) DAPI included images of *WT* and *Mcm5*^*A7*^ in Fig 4A, 4B and 4C, respectively, to demonstrate that additional CID foci are of neighboring nuclei. d. DAPI included images of meiotic nuclei with 1 359-bp focus (*WT*, top panel) and 2 359-bp foci (*Mcm5*^*A7*^, bottom panel) from Fig 4D. Scale bars = 1 μm. Contrast and brightness of all images were adjusted for clarity.
(TIF)

S3 Fig Arm cohesion in *Mcm5*^*A7*^ mutants. a. Representative images quantified in Fig 5B and 5C. Scale bar = 1 μm. Magenta: SMC1, Greed: CID. Images are of whole mount germaria. b-d. Schematic of possible outcomes in *X* ring:rod assay (modified from Webber, Howard, and Bickel 2004). b. In the absence of crossover events, meiotic products will be 2 ring chromosomes and 2 rod chromosomes, yielding viable progeny that inherit a ring chromosome or a rod chromosome at 1:1. c. In presence of inter-homolog crossing over, meiotic products will be 1 dicentric chromosome, 1 ring chromosome, and 1 rod chromosome. Progeny with a dicentric chromosome will not be viable, thus viable progeny will inherit a ring or a rod chromosome at 1:1. d. In the occurrence of inter-sister crossing over, the two ring chromosomes will form a dicentric ring chromosome, which will not yield viable progeny. Viable progeny in the presence of high inter-sister crossing over will inherit a ring or a rod chromosome at 0:2, lowering the overall ring to rod ratio.
(TIF)

S4 Fig SMC1 overexpression in *Mcm5*^*A7*^ mutants. a. Representative images of *nos>Smc1*, *Mcm5*^*A7*^ meiotic nuclei at meiotic onset, quantified in Fig 6A and 6B. Crossovers levels on Chromosome *X* in *WT* (*n* = 2179, 62.8cM) [54] and *Mcm5*^*A7*^ (*n* = 2743, 3.8 cM), similar to levels previously reported [1]. These data show that the crossover defect severity in *Mcm5*^*A7*^ mutants is chromosome-specific. Due to genetics of the SMC1 transgene, we were unable to test *nos>Smc1; Mcm5*^*A7*^ crossover levels on the *X*. Data are represented as mean ± 95% CI. See S4 Table for complete crossover dataset. c. Left: NDJ of the *X* chromosome in *WT* (0.07%, *n* = 3034) and controls *nos>Smc1; Mcm5*^*Df/+*^ (0.16%, *n* = 1273) and *Mcm5>Mcm*^*WT*^; *Mcm5*^*A7*^ (0.26%, *n* = 753). Right: NDJ of *rec*^*1/2*^ (19.1%, *n* = 1563), and *nos>Smc1*, *rec*^*1/2*^ (24.1%, *n* = 1187) to demonstrate that SMC1 overexpression NDJ rescue is specific to *Mcm5*^*A7*^. Data are represented as mean ± 95% CI.
(TIF)

S5 Fig Pairing effects of recombination and centromere mutations in *Mcm5*^*A7*^ mutants. a. Quantification of percent paired and unpaired *X* loci in *Mcm5*^*A7*^ and *mei-P22*, *Mcm5*^*A7*^ in early pachytene and mid-pachytene nuclei (germarium regions 2 and 3). 46% of examined nuclei are unpaired in *Mcm5*^*A7*^ (total *n* = 129), and 42% of examined nuclei are unpaired in *mei-P22*, *Mcm5*^*A7*^ (total *n* = 55). b. Quantification of CID foci in early pachytene nuclei in *WT* (*n* = 65), *Mcm5*^*A7*^ (*n* = 94), and *cal1*^*2k32*^*/cal1*^*+*^, *Cenp-C*^*pr141*^/Cenp-C^+^ (*n* = 49). ***p < 0.0001, unpaired T-test. Data are represented as mean ± SD. c. Quantification of CID foci in early pachytene nuclei in *WT* (*n* = 16), *Mcm5*^*A7*^ (*n* = 19), *cal1*^*2k32*^*/cal1*^*+*^, *Cenp-C*^*pr141*^/Cenp-C^+^ (*n* = 8), *cal1*^*2k32*^*/cal1*^*+*^, *Cenp-C*^*Df*^/Cenp-C^+^ (*n* = 20), *cal1*^*2k32*^*/cal1*^*+*^, *Cenp-C*^*Z3-4375*^/Cenp-C^+^ (*n* = 18). **p = 0.0042, *n*.*s*. = 0.5 and 0.9, respectively, unpaired T-test. Data are represented as mean ± SD. Data for *cal1*^*2k32*^*/cal1*^*+*^, *Cenp-C*^*Df*^/Cenp-C^+^ and *cal1*^*2k32*^*/cal1*^*+*^, *Cenp-C*^*Z3-4375*^/Cenp-C^+^ are previously published [38]. d. Quantification of percent paired and unpaired *X* loci in *Cenp-C*^*pr141*^/Cenp-C^+^ early and mid-pacytene nuclei (*n* = 70).
(TIF)

S1 Table Complete inter-sister recombination dataset. Complete Ring:Rod dataset, as shown in Fig 5. *Adjusted females: Assuming male and female NDJ are equal, we subtract the amount of male NDJ (exceptional progeny) from the *y* Normal female progeny; in these *y* females, one cannot distinguish between *y/y* female versus a *y/y/Y* female. *Ord*^*10*^ exceptional females were distinguishable due to an additional phenotypic marker. **Adjusted total: Adjusted Normal Females + Normal Males. Total Ring^#^: Adjusted Normal Females Ring + Normal Males Ring. Total Rod^##^: Adjusted Normal Females Rod + Normal Males Rod. Ratio^$^: Total ring / total rod.
(XLSX)

S2 Table Recombination dataset for Chromosome *2*L. Recombination events across Chromosome *2*L in progeny of *WT*, *Mcm5*^*A7*^, and *nos>Smc1*, *Mcm5*^*A7*^ mothers.
(XLSX)

S3 Table Complete *X-*NDJ dataset. Total normal and exceptional progeny from experimental and control lines.
(XLSX)

S4 Table Recombination dataset for *X* Chromosome. Recombination events across *X* Chromosome in progeny of *WT*, *Mcm5*^*A7*^, and *nos>Smc1*, *Mcm5*^*A7*^ mothers.
(XLSX)

S1 Movie Rotation of *WT* meiotic nucleus shown in Fig 2B. Meiotic nucleus demonstrating full length tracts of C(3)G (magenta) and localization of *X-*homologs (*X*-probes, green) in *WT*.
(MOV)

S2 Movie Rotation of *Mcm5*^*A7*^ meiotic nucleus shown in Fig 2B. Meiotic nucleus demonstrating full length tracts of C(3)G (magenta) and localization of *X-*homologs (*X*-probes, green) in *Mcm5*^*A7*^.
(MOV)

S3 Movie Dynamics of centromere clusters in 8-cell cyst nuclei in *WT*. Time lapse microscopy (spinning disc) expressing the centromere CID::RFP (magenta) and fusome marker Par-1::GFP (driven by the *nanos* promoter) (green). Frames were taken every 10 seconds.
(MP4)

S4 Movie Dynamics of centromere clusters in 8-cell cyst nuclei in *Mcm5*^*A7*^. Time lapse microscopy (spinning disc) expressing the centromere CID::RFP (magenta) and fusome marker Par-1::GFP, (driven by the *nanos* promoter) (green). Frames were taken every 10 seconds.
(MP4)

S5 Movie Dynamics of one centromere cluster in one 8-cell cyst nucleus in *WT*. Time lapse microscopy (spinning disc) expressing the centromere CID::RFP (magenta) in one nucleus within an 8-cell cyst (dotted circle). Frames were taken every 10 seconds.
(MP4)

S6 Movie Dynamics of one centromere cluster in one 8-cell cyst nucleus in *Mcm5*^*A7*^. Time lapse microscopy (spinning disc) expressing the centromere CID::RFP (magenta) in one nucleus within an 8-cell cyst (dotted circle). Frames were taken every 10 seconds.
(MP4)
