# Supplementary figures and images for: Centromeric SMC1 promotes centromere clustering and stabilizes meiotic homolog pairing
Source: PLoS Genet. 2019 Oct 14;15(10):e1008412. doi: 10.1371/journal.pgen.1008412 (PMC6812850; doi:10.1371/journal.pgen.1008412)

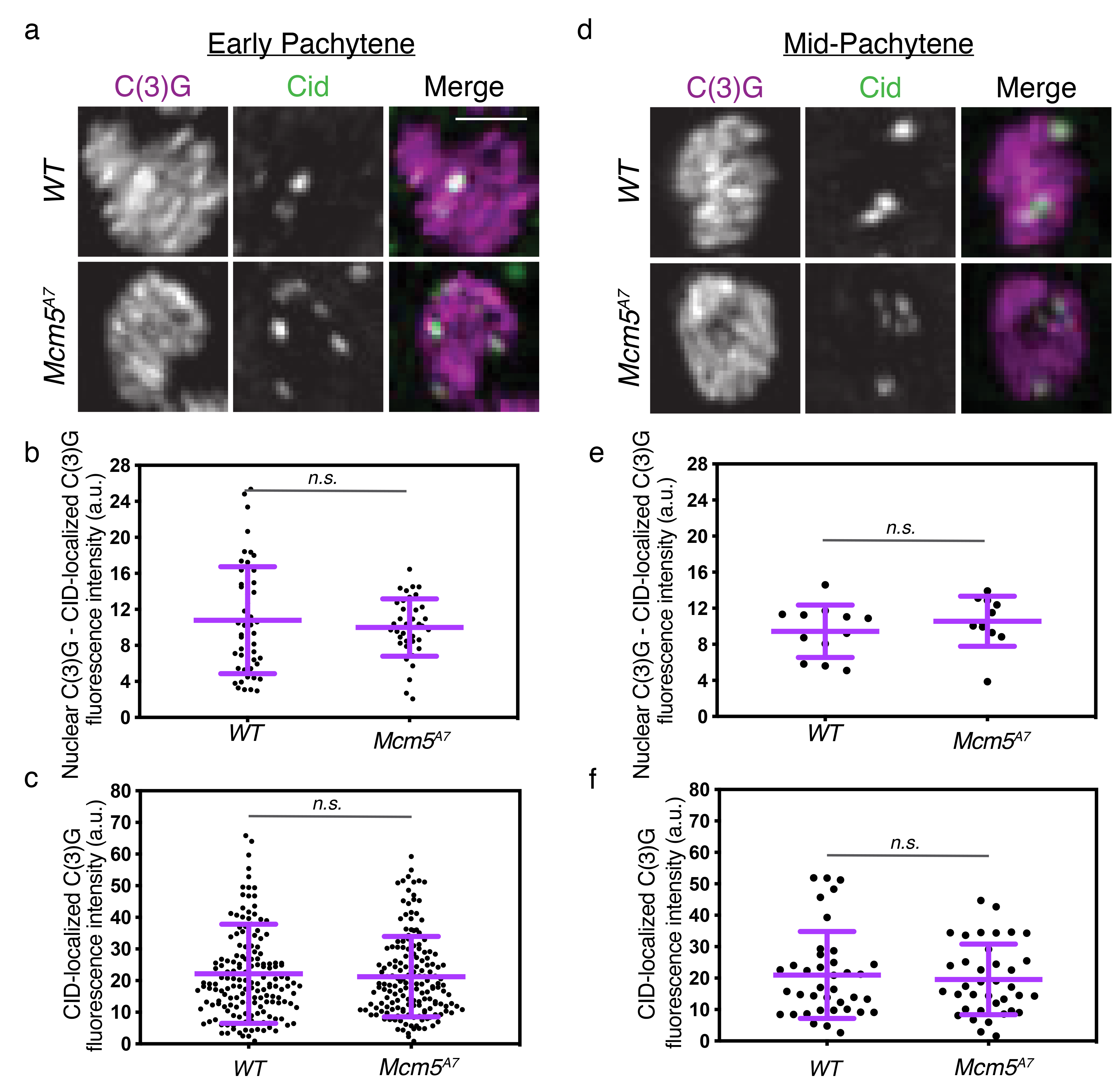

Supplement: S1 Fig — a. Representative images of WT and Mcm5A7 meiotic nuclei in whole mount germaria that were quantified in b., c., and Fig 2E examining C(3)G (magenta) and CID (green) in early pachytene. b. Quantification of C(3)G signal at the centromere (CID) in WT and Mcm5A7 early pachytene nuclei. p = 0.4327, unpaired T-test. Data are represented as mean ± SD. c. Quantification of C(3)G signal at chromosome arm in WT and Mcm5A7 early pachytene nuclei. p = 0.6358, unpaired T-test. Data are represented as mean ± SD. d. Representative images of WT and Mcm5A7 meiotic nuclei of whole mount germaria that were quantified in e., f., and Fig 2F examining C(3)G (magenta) and CID (green) at mid-pachytene. e. Quantification of C(3)G signal at the centromere (CID) in WT and Mcm5A7 mid-pachytene nuclei. p = 0.3615, unpaired T-test. Data are represented as mean ± SD. f. Quantification of C(3)G signal at chromosome arms in WT and Mcm5A7 mid-pachytene nuclei. p = 0.5489, unpaired T-test. Data are represented as mean ± SD. (TIF) [file pgen.1008412.s001.tif]

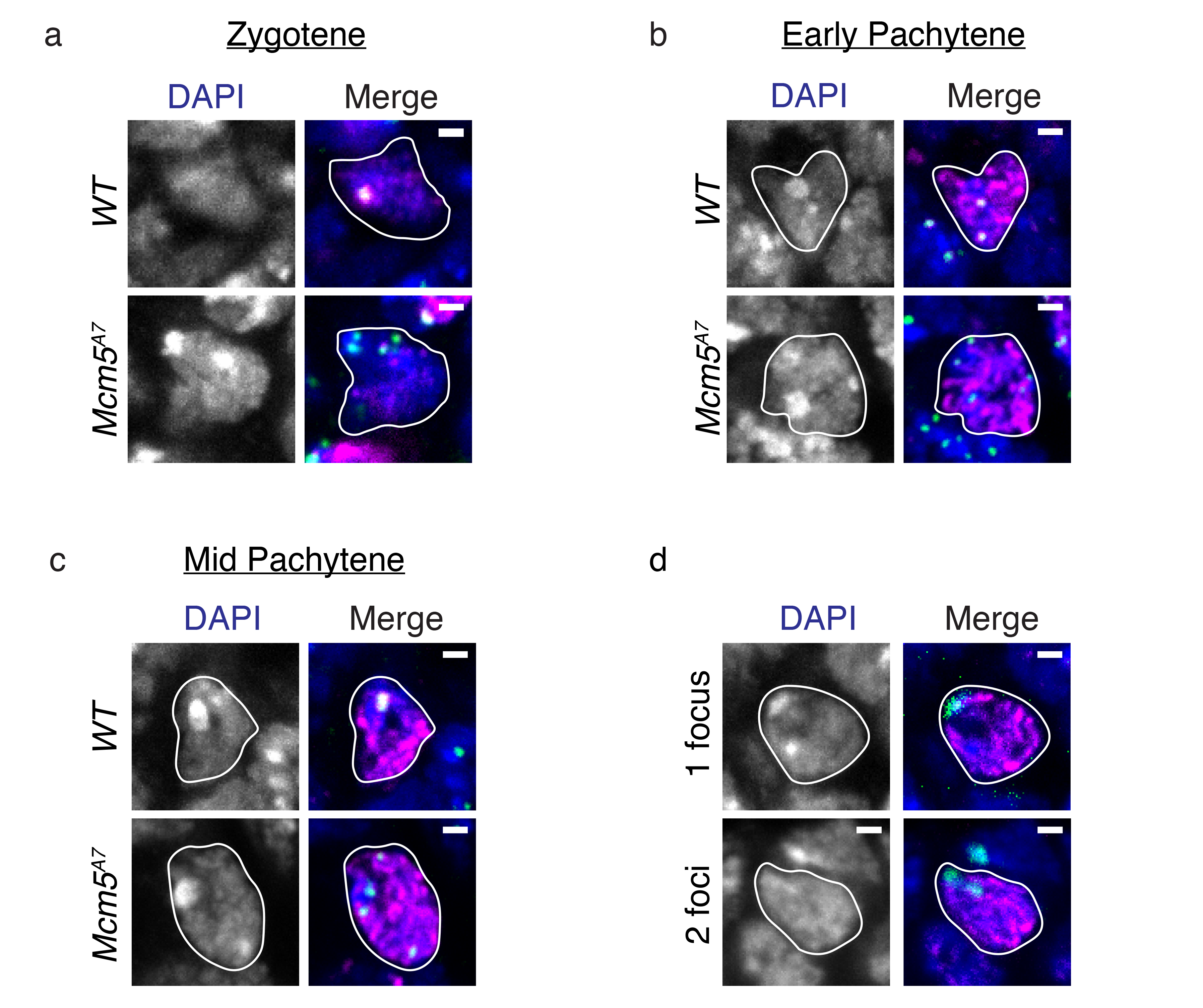

Supplement: S2 Fig — (a, b, c) DAPI included images of WT and Mcm5A7 in Fig 4A, 4B and 4C, respectively, to demonstrate that additional CID foci are of neighboring nuclei. d. DAPI included images of meiotic nuclei with 1 359-bp focus (WT, top panel) and 2 359-bp foci (Mcm5A7, bottom panel) from Fig 4D. Scale bars = 1 μm. Contrast and brightness of all images were adjusted for clarity. (TIF) [file pgen.1008412.s002.tif]

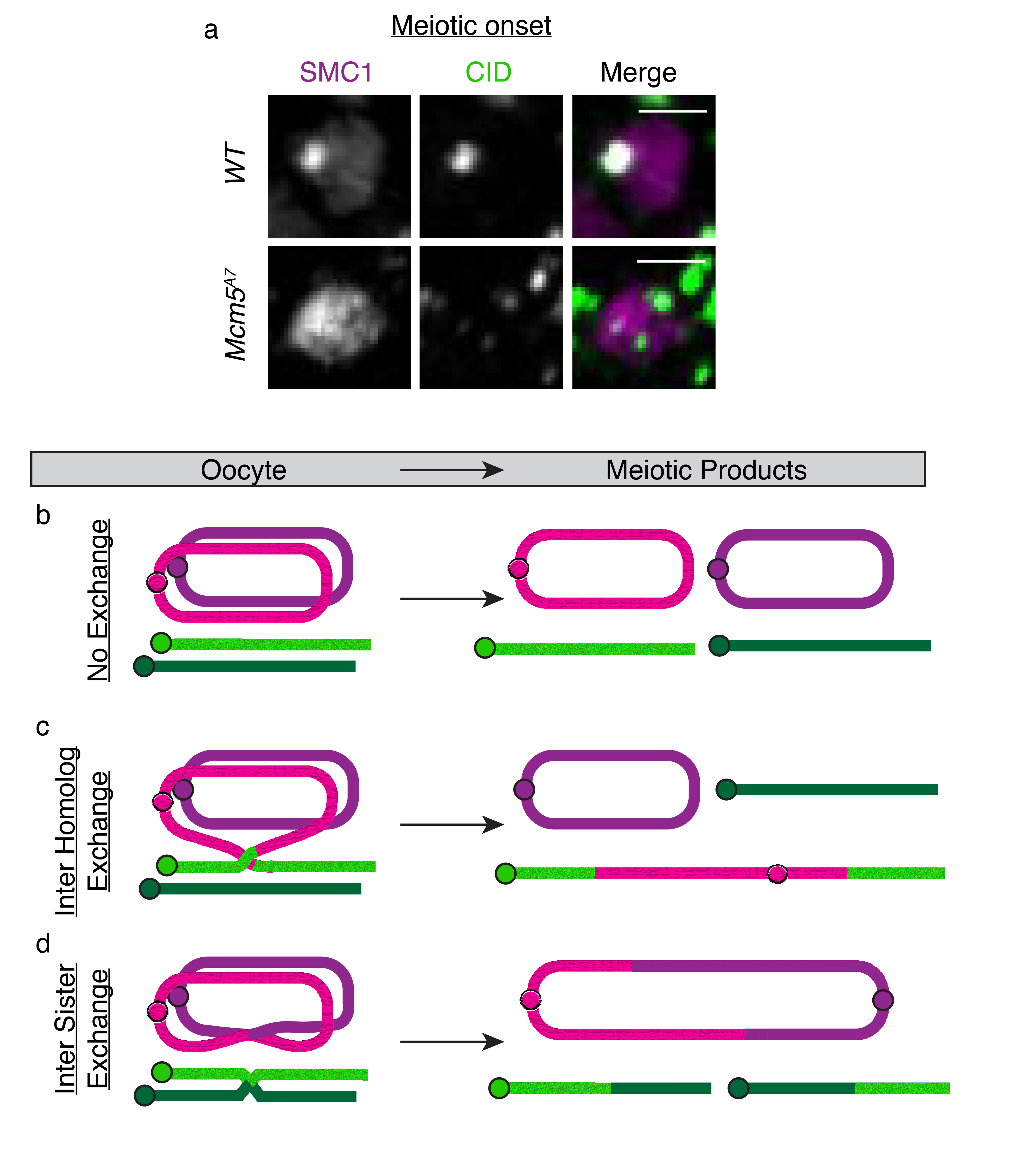

Supplement: S3 Fig — a. Representative images quantified in Fig 5B and 5C. Scale bar = 1 μm. Magenta: SMC1, Greed: CID. Images are of whole mount germaria. b-d. Schematic of possible outcomes in X ring:rod assay (modified from Webber, Howard, and Bickel 2004). b. In the absence of crossover events, meiotic products will be 2 ring chromosomes and 2 rod chromosomes, yielding viable progeny that inherit a ring chromosome or a rod chromosome at 1:1. c. In presence of inter-homolog crossing over, meiotic products will be 1 dicentric chromosome, 1 ring chromosome, and 1 rod chromosome. Progeny with a dicentric chromosome will not be viable, thus viable progeny will inherit a ring or a rod chromosome at 1:1. d. In the occurrence of inter-sister crossing over, the two ring chromosomes will form a dicentric ring chromosome, which will not yield viable progeny. Viable progeny in the presence of high inter-sister crossing over will inherit a ring or a rod chromosome at 0:2, lowering the overall ring to rod ratio. (TIF) [file pgen.1008412.s003.tif]

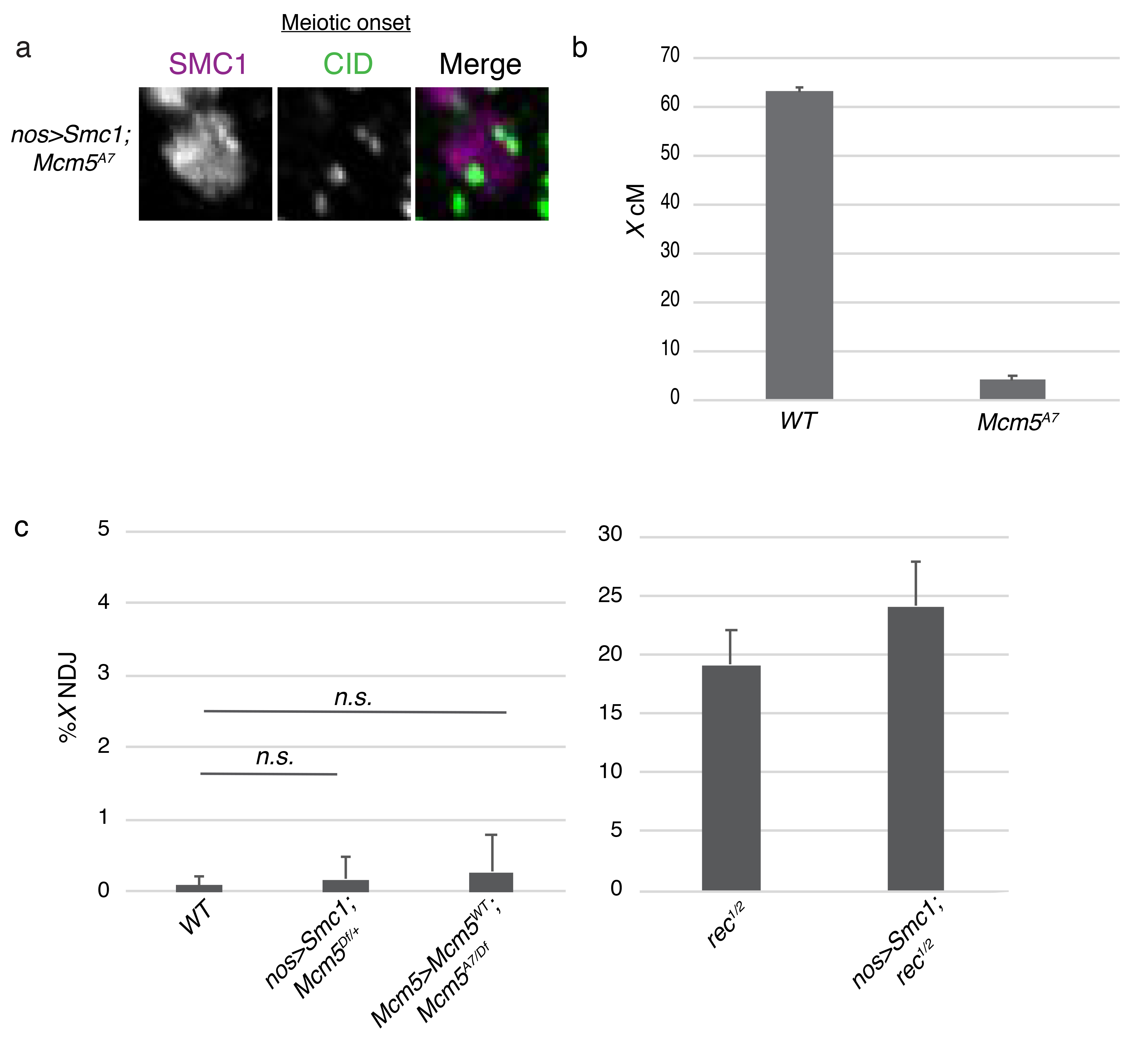

Supplement: S4 Fig — a. Representative images of nos>Smc1, Mcm5A7 meiotic nuclei at meiotic onset, quantified in Fig 6A and 6B. Crossovers levels on Chromosome X in WT (n = 2179, 62.8cM) [54] and Mcm5A7 (n = 2743, 3.8 cM), similar to levels previously reported [1]. These data show that the crossover defect severity in Mcm5A7 mutants is chromosome-specific. Due to genetics of the SMC1 transgene, we were unable to test nos>Smc1; Mcm5A7 crossover levels on the X. Data are represented as mean ± 95% CI. See S4 Table for complete crossover dataset. c. Left: NDJ of the X chromosome in WT (0.07%, n = 3034) and controls nos>Smc1; Mcm5Df/+ (0.16%, n = 1273) and Mcm5>McmWT; Mcm5A7 (0.26%, n = 753). Right: NDJ of rec1/2 (19.1%, n = 1563), and nos>Smc1, rec1/2 (24.1%, n = 1187) to demonstrate that SMC1 overexpression NDJ rescue is specific to Mcm5A7. Data are represented as mean ± 95% CI. (TIF) [file pgen.1008412.s004.tif]

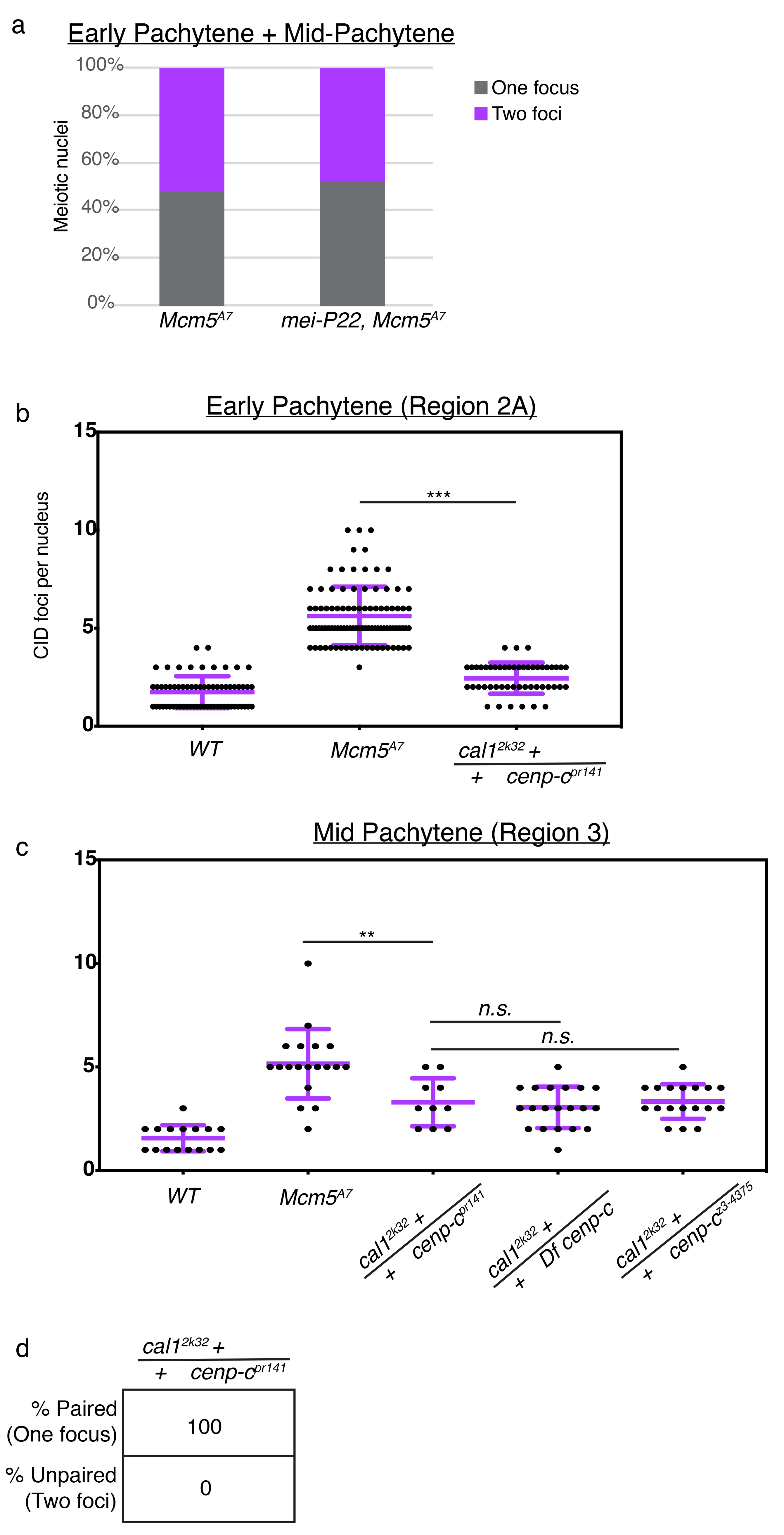

Supplement: S5 Fig — a. Quantification of percent paired and unpaired X loci in Mcm5A7 and mei-P22, Mcm5A7 in early pachytene and mid-pachytene nuclei (germarium regions 2 and 3). 46% of examined nuclei are unpaired in Mcm5A7 (total n = 129), and 42% of examined nuclei are unpaired in mei-P22, Mcm5A7 (total n = 55). b. Quantification of CID foci in early pachytene nuclei in WT (n = 65), Mcm5A7 (n = 94), and cal12k32/cal1+, Cenp-Cpr141/Cenp-C+ (n = 49). ***p < 0.0001, unpaired T-test. Data are represented as mean ± SD. c. Quantification of CID foci in early pachytene nuclei in WT (n = 16), Mcm5A7 (n = 19), cal12k32/cal1+, Cenp-Cpr141/Cenp-C+ (n = 8), cal12k32/cal1+, Cenp-CDf/Cenp-C+ (n = 20), cal12k32/cal1+, Cenp-CZ3-4375/Cenp-C+ (n = 18). **p = 0.0042, n.s. = 0.5 and 0.9, respectively, unpaired T-test. Data are represented as mean ± SD. Data for cal12k32/cal1+, Cenp-CDf/Cenp-C+ and cal12k32/cal1+, Cenp-CZ3-4375/Cenp-C+ are previously published [38]. d. Quantification of percent paired and unpaired X loci in Cenp-Cpr141/Cenp-C+ early and mid-pacytene nuclei (n = 70). (TIF) [file pgen.1008412.s005.tif]
